# Cell-matrix adhesion controls Golgi organization and function through Arf1 activation in anchorage-dependent cells

**DOI:** 10.1242/jcs.215855

**Published:** 2018-08-17

**Authors:** Vibha Singh, Chaitanya Erady, Nagaraj Balasubramanian

**Affiliations:** Indian Institute of Science Education and Research, Dr. Homi Bhabha Road, Pune, Maharashtra 411008, India

**Keywords:** Golgi, Integrin, Adhesion, Arf1, Dynein, Glycosylation

## Abstract

Cell-matrix adhesion regulates membrane trafficking controlling anchorage-dependent signaling. While a dynamic Golgi complex can contribute to this pathway, its regulation by adhesion remains unclear. Here we report that loss of adhesion dramatically disorganized the Golgi in mouse and human fibroblast cells. Golgi integrity is restored rapidly upon integrin-mediated re-adhesion to FN and is disrupted by integrin blocking antibody. In suspended cells, the cis, cis-medial and trans-Golgi networks differentially disorganize along the microtubule network but show no overlap with the ER, making this disorganization distinct from known Golgi fragmentation. This pathway is regulated by an adhesion-dependent reduction and recovery of Arf1 activation. Constitutively active Arf1 disrupts this regulation and prevents Golgi disorganization due to loss of adhesion. Adhesion-dependent Arf1 activation regulates its binding to the microtubule minus-end motor protein dynein to control Golgi reorganization, which is blocked by ciliobrevin. Adhesion-dependent Golgi organization controls its function, regulating cell surface glycosylation due to loss of adhesion, which is blocked by constitutively active Arf1. This study, hence, identified integrin-dependent cell-matrix adhesion to be a novel regulator of Arf1 activation, controlling Golgi organization and function in anchorage-dependent cells.

This article has an associated First Person interview with the first author of the paper.

## Abstract (precis)

**Summary:** Integrin-dependent cell-matrix adhesion activates Arf1, which then recruits dynein to regulate Golgi organization and function along the microtubule network.

## INTRODUCTION

Cell-matrix adhesion is a vital regulator of many cellular processes (Berrier and Yamada, 2007; Caswell et al., 2009; Danen and Yamada, 2001) and disease conditions (Desgrosellier and Cheresh, 2010; Wu and Reddy, 2012). A role for integrin-mediated adhesion in regulating membrane trafficking is seen to affect membrane order (Gaus et al., 2006), receptor mobility and activation to drive anchorage-dependent signaling (Balasubramanian et al., 2007b, 2010; Pawar et al., 2016). Loss of adhesion dramatically turns off this signaling, which then recovers upon re-adhesion to the matrix (del Pozo et al., 2004). Cancer cells deregulate this trafficking to become anchorage-independent, supporting oncogenic transformation (Guo and Giancotti, 2004; Schwartz, 1997). An important mediator of trafficking and processing of membrane lipids and proteins in the cell is the Golgi complex (Bankaitis et al., 2012; Rodriguez-Boulan and Müsch, 2005). Known to be a dynamic structure, the organization, and positioning of the Golgi in the cell is vital to directional trafficking and secretion during processes, such as cell polarization, migration and division (Wilson et al., 2011; Yadav and Linstedt, 2011). Adhesion of cells on micropatterned surfaces to mimic a polarized phenotype is seen to affect the localization of the Golgi relative to the centrosome and nucleus (Thery et al., 2006).

The organization of the Golgi complex is subject to many variables, and directly contributes to Golgi inheritance and function (Shorter and Warren, 2002). In mammalian cells, the Golgi is made up of a series of flattened cisternal stacks that are not homogeneous, and contain different resident proteins and enzymes that allow the Golgi to be structurally divided into cis, medial and trans regions (Papanikou and Glick, 2014; Shorter and Warren, 2002). The cis-Golgi receives newly synthesized proteins and lipids from the endoplasmic reticulum (ER), which are processed as they move through the Golgi and exit from the trans-Golgi region (Glick and Luini, 2011). The most-trans regions of the Golgi are continuous with a tubular, branching and reticulating compartment termed the trans-Golgi network (TGN) (Klumperman, 2011), which sorts and delivers proteins and lipids to their cellular destinations. The Golgi typically has a perinuclear localization around the microtubule organizing center (MTOC) (Hurtado et al., 2011), its assembly and maintenance being controlled by both microtubules (Gurel et al., 2014) and the actin cytoskeleton (Zilberman et al., 2011). A subset of the microtubule network is nucleated at the Golgi and thought to play a role in assembling Golgi mini stacks into a distinct Golgi ribbon (Zhu and Kaverina, 2013). They are further seen to bind membrane-stacking proteins, such as GRASP65 and GRASP5, that differentially localize to the distinct Golgi compartments (Vinke et al., 2011). The Golgi complex undergoes dramatic fragmentation during cell division (Corda et al., 2012), apoptosis (Mukherjee et al., 2007) and pathological conditions, such as neuronal degeneration (Nakagomi et al., 2008) and cancer (Petrosyan, 2015). This fragmentation is, at times, irreversible, i.e. during apoptosis (Mukherjee et al., 2007), and sometimes reversible, as seen during cell division (Corda et al., 2012).

The small GTPase Arf1, acts as a main regulator of Golgi organization and function. Upon its activation, Arf1 associates with Golgi membranes and is released from the Golgi into the cytosol upon its inactivation (Donaldson et al., 2005). This regulation of Arf1 activation is mediated by guanine nucleotide exchange factors (GEFs) and GTPase-activating proteins (GAPs) (D'Souza-Schorey and Chavrier, 2006). Arf1 binds and/or regulates adaptor, stacking and structural proteins as well as lipid-modifying enzymes on the Golgi membrane. It is, hence, able to influence multiple aspects of Golgi organization and function in cells (D'Souza-Schorey and Chavrier, 2006). Integrin-mediated adhesion regulates the Arf family GTPase Arf6 through cytohesin 2 (hereafter referred to as ARNO) (Lee et al., 2014; Pawar et al., 2016), to control adhesion-dependent membrane exocytosis and signaling (Balasubramanian et al., 2007b, 2010; Pawar et al., 2016). Adhesion is also seen to control cell polarization and migration (Cox et al., 2001; Huttenlocher and Horwitz, 2011), cell cycle progression (Ben-Ze'ev and Raz, 1981; Schwartz and Assoian, 2001) and apoptosis (Meredith and Schwartz, 1997), all of which are seen to be associated with changes in Golgi integrity. A role for integrin-mediated adhesion in regulating Golgi organization and function remains largely unexplored. In this study, we tested for and identified the presence of such a regulatory pathway that drives Golgi organization and function in anchorage-dependent cells.

## RESULTS

### Cell adhesion regulates Golgi organization

**Fig. 1.**
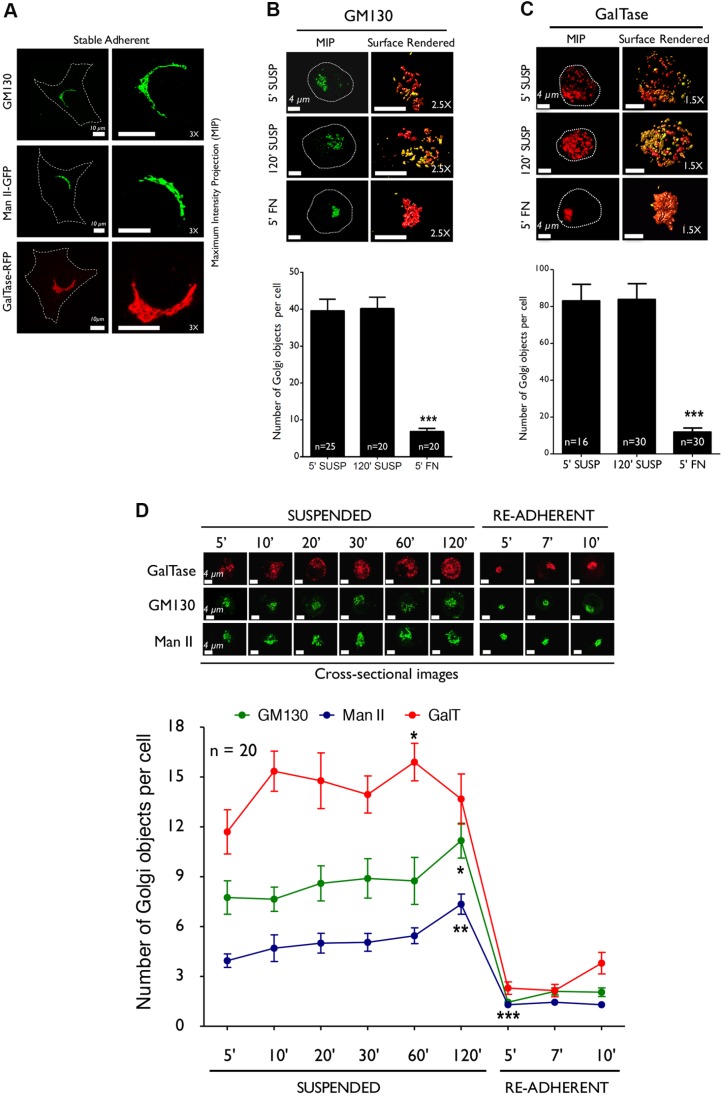
**Adhesion regulates Golgi organization.** (A) Stable adherent WT-MEFs were immunostained for cis-Golgi marker (GM130), transfected with the cis-medial Golgi marker (ManII) or the trans-Golgi marker (GalTase). Representative MIP (left) and 3× magnified images (right) of cells are shown. (B) Representative MIP (left) and de-convoluted surface-rendered images of non-transfected WT-MEFs that had just been detached (5′ SUSP), had been held in suspension for 120 min (120′ SUSP) and had been re-plated on FN (2 µg/ml) for 5 min (5′ FN) were stained for GM130. (C) GalTase-expressing WT-MEFs had been processed similarly. Representative MIP (left) and de-convoluted surface-rendered images (right) (magnified 2.5× for GM130 and 1.5× for GalTase) are shown. Graph represents discontinuous cis-Golgi (GM130) and trans-Golgi objects (GalTase) per cell as mean±s.e. of 16–30 cells from 3 independent experiments. (D) WT-MEFs expressing GalTase (top) or ManII (bottom), or had been immunostained with GM130 (middle) were held in suspension for increasing time points (5′, 10′, 20′, 30′, 60′, 120′ SUSPENDED) or re-adhered on FN (5′, 7′, 10′ RE-ADHERENT). Representative MIP cross-sectional images for each Golgi marker at every time point are shown. The graph represents mean±s.e. for Golgi objects in cross-sectional images of 20 cells from 2 independent experiments. Scale bars: 10 µm (A), 4 µm (B–D). Statistical analysis was done using Mann–Whitney U test (*P<0.05, **P<0.001, ***P<0.0001).

To test whether cell-matrix adhesion can affect Golgi organization, stable adherent wild-type mouse embryonic fibroblasts (WT-MEFs) were grown under low-serum conditions for 12 h to suppress growth factor signaling, detached and held in suspension (with methylcellulose), and re-plated on fibronectin (FN). The effect loss of adhesion and re-adhesion have upon Golgi organization in these cells was tested by using cis [golgin subfamily A member 2 (GM130); Nakamura, 2010], cis-medial [GFP-tagged mannosidase 2 (ManII-GFP); Mardones et al., 2006] and trans-Golgi markers [RFP-tagged galactosyltransferase (GalTase-RFP); Barbero et al., 2002]. They were chosen to compare the relative effects adhesion has on individual Golgi compartments. Cells labeled with Golgi marker constructs were chosen for low to moderate expression to rule out effects overexpression might have on the Golgi phenotype. Following serum deprivation, stable adherent WT-MEFs had an intact Golgi (Fig. 1A). Cells that had been in suspension for ∼5 min (5′ SUSP; detachment of cells took ∼5 min to carry out), began to disorganize their cis-Golgi (GM130) (Fig. 1B) and, more prominently, their trans-Golgi networks (GalTase) (Fig. 1C), both further dispersing as cells were held in suspension for 120 min (120′ SUSP). Re-adhesion on FN-coated coverslips for 5 min did not significantly affect the cell shape or volume (Fig. S1A) but dramatically restored cis- (Fig. 1B), cis-medial (Fig. S1B) and trans-Golgi organization (Fig. 1C). By using the prominent trans-Golgi phenotype, the distribution of WT-MEFs with visibly organized versus disorganized Golgi complexes confirmed suspended cells to have a majorly disorganized phenotype, which is reversed upon re-adhesion (Fig. S1C).

This reorganization of the Golgi in WT-MEFs is further reflected in the significant reduction in the number of discontinuous cis and trans-Golgi objects (calculated from confocal *z*-stacks de-convoluted using the Huygens Professional software) in re-adherent relative to suspended cells (see graphs in Fig. 1B,C). The numbers of trans-Golgi object calculated in that manner were almost double compared with those seen for the cis-Golgi, reflecting their more-prominent disorganization upon loss of adhesion. The net trans-Golgi volume showed a significant increase upon the loss of adhesion, which was restored upon re-adhesion (Fig. S1D, right panel). The volume of the cis-Golgi, however, did not change significantly (Fig. S1D, left panel), suggesting that the extent of disorganization for cis and trans-Golgi upon loss of adhesion is distinctly different.

These experiments were all carried out using cells detached with trypsin, which might have affected cell surface proteins and influenced the phenotype observed. To rule out this possibility we used the trypsin replacement Accutase (Bajpai et al., 2008) to detach cells and found the adhesion-dependent regulation of the Golgi (detected with ManII and GalTase) to be comparable to that using trypsin (Fig. S1E). We also evaluated whether this regulation is unique to WT-MEFs by testing the behavior using cells of the human fibroblast cell line BJ that express ManII-GFP and GalTase-RFP. The Golgi in serum-deprived stable adherent BJ cells was found to be intact (Fig. S1F), rapidly disorganizing upon loss of adhesion and reorganizing upon re-adhesion to FN (Fig. S1G,H). The trans-Golgi (GalTase) in BJ cells was also visibly more disorganized than the cis-medial-Golgi (ManII), with a significantly higher number of discontinuous objects (see graphs in Fig. S1G,H). Upon re-adhesion, these objects decreased significantly in number for both the cis-medial and the trans-Golgi, reflecting their rapid reorganization (see graphs in Fig. S1G,H). The adhesion-dependent regulation of Golgi organization is, hence, conserved across anchorage-dependent mouse and human fibroblasts.

To further establish the Golgi phenotype described above in WT-MEFs, we looked at the behavior of an additional cis-Golgi marker [the general vesicular transport factor p115 (p115); Dejgaard et al., 2007] and trans-Golgi network (TGN) markers [syntaxin-6 (Reverter et al., 2014) and RFP-tagged TGN protein 2 (TGN38-RFP); De Matteis and Luini, 2008]. Emerging from the trans-Golgi the TGN is known to undergo dynamic changes that affect protein sorting and trafficking from the Golgi (De Matteis and Luini, 2008; Rodriguez-Boulan and Müsch, 2005). Upon loss of adhesion, syntaxin-6- and TGN38-labeled TGN showed a significantly more disorganized phenotype than the p115-labeled cis-Golgi, all 3 markers rapidly reorganizing upon re-adhesion to FN (Fig. S1I–K). Together, these results confirm the differential disorganization of the cis-Golgi relative to the trans-Golgi and TGN upon loss of adhesion. The regulation of the TGN is likely to further contribute to the altered behavior of the Golgi upon loss of adhesion.

### Kinetics and relative organization of cis-, cis-medial and trans-Golgi networks in WT-MEFs

To better understand the adhesion-dependent regulation of Golgi compartments, we tested the kinetics of disorganization for the cis (GM130), cis-medial (ManII) and trans-Golgi (GalTase) by using cross-sectional images. Detachment of cells (5′ SUSP) was seen to disorganize all 3 markers, their individual object numbers increasing in suspension only marginally over time (Fig. 1D). Again, the trans-Golgi was more disorganized than the cis or cis-medial-Golgi. Re-adhesion of cells caused all three Golgi markers to be rapidly reorganized, with a significant decrease in discontinuous object numbers. This change is retained as the cells attach and spread during the investigation period of 10 min (see graph in Fig. 1D).

**Fig. 2.**
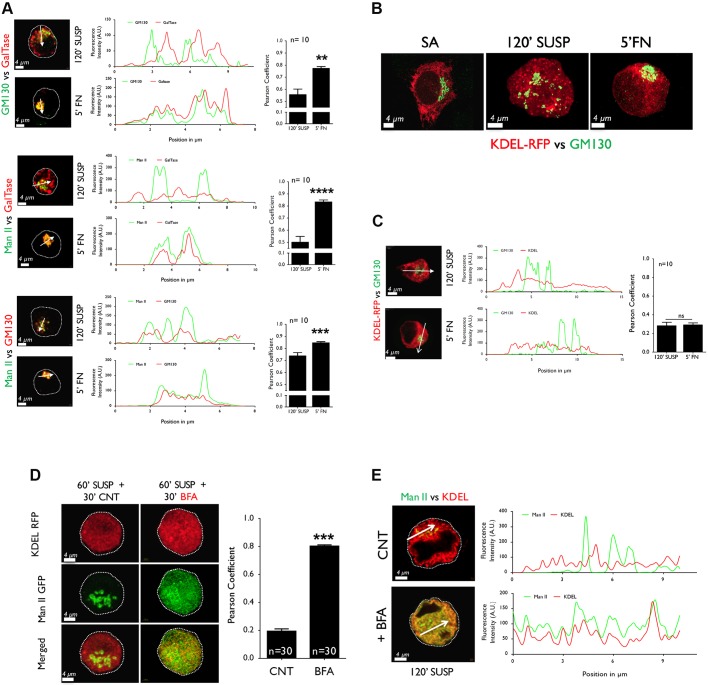
**Adhesion-dependent regulation of Golgi compartments relative to each other and the ER.** (A) Cells expressing or immunostained with a cis (GM130) and trans (GalTase) (top), cis-medial (ManII) and trans (GalTase) (middle), or ManII and GM130 (bottom) were compared in suspended (120′ SUSP) and re-adherent cells (5′ FN). Line plot of fluorescence intensity in representative suspended and re-adherent cell was measured and plotted. Graph represents Pearson's coefficient of cells as mean±s.e. from 10 independent cells in 2 independent experiments. (B) Representative images of WT-MEFs expressing the ER marker (KDEL-RFP). Shown is immunostaining with GM130 (cis-Golgi) of stable adherent (SA), 120′ SUSP and 5′ FN WT-MEFs. (C) Line plot of fluorescence intensity in representative suspended and re-adherent cell was measured and plotted. Graph represents Pearson's coefficient of cells as mean±s.e. from 10 independent cells in 2 independent experiments. (D) Cells expressing ER marker (KDEL-RFP) and cis-medial Golgi (ManII-GFP) suspended for 60′ were treated with MeOH (60′SUSP+ 30′CNT) or BFA (60′SUSP+ 30′BFA) for 30 min. Representative MIP and merged *z*-stack of ER, cis-medial Golgi is shown. Graph represents Pearson's coefficient as mean±s.e. of 30 cells from 3 independent experiments. (E) Line plot of fluorescence intensity in representative CNT and BFA treated cell were measured and plotted. Scale bar in all images is set at 4 µm. Statistical analysis was done using Mann–Whitney U test (**P<0.01, ****P<0.00001).

We further looked at the localization of the cis, cis-medial and trans-Golgi relative to each other in suspended and re-adherent cells. In comparison, the cis versus trans-Golgi (GM130 vs GalTase; see top panel Fig. 2A) and cis-medial versus trans Golgi (ManII versus GalTase; see middle panel Fig. 2A) show little overlap in suspended cells, but this is restored as the Golgi reorganizes upon re-adhesion. In suspended cells the cis versus cis-medial Golgi (GM130 vs ManII) stay in close proximity, their overlap increasing marginally but significantly upon re-adhesion (see bottom panel Fig. 2A). This is also reflected in line plots for these marker combinations in suspended and re-adherent cells, and their Pearson's coefficient (see graphs in Fig. 2A). These observations show that the change in organization for each Golgi compartment was triggered simultaneously (Fig. 1D), but had different effects (Fig. 2A).

Knowing that fragmentation of the Golgi is triggered during mitosis (Altan-Bonnet et al., 2006) and brefeldin A (BFA) treatment (Lippincott-Schwartz et al., 1989), we asked whether loss of adhesion affects the Golgi-ER overlap. The distinct tubular organization of the ER detected by using RFP-tagged to the KDEL target sequence (KDEL-RFP; Altan-Bonnet et al., 2006) in stable adherent cells is less prominent in suspended cells, although the ER continues to occupy the bulk of the cell cytosol with a more punctate organization in suspended and re-adherent cells (Fig. 2B). The cis-Golgi (GM130) in suspended and re-adherent cells fail to show any significant overlap with the ER (KDEL-RFP), suggesting its disorganized phenotype to be distinct from known Golgi fragmentation (Lippincott-Schwartz et al., 1989). This is confirmed by comparing their line plots and Pearson's coefficient (Fig. 2C). We further tested whether, in suspended cells, the Golgi can be artificially fragmented by treating cells with BFA and inhibiting Arf1 (Fig. S2A) (Fujiwara et al., 1988). Suspended WT-MEFs (60′ SUSP) with a disorganized cis-medial Golgi – when incubated with BFA for an additional 30 min – dramatically fragment and fall back into the ER (Fig. 2D), which is reflected in their line plots (Fig. 2E) and Pearson's coefficient (see graph in Fig. 2D). Taken together, these results confirm adhesion-dependent disorganization of the Golgi to be distinctly different from known Golgi fragmentation, leading us to ask how this pathway is regulated in anchorage-dependent fibroblasts.

### Integrin-mediated adhesion regulates Golgi organization

**Fig. 3.**
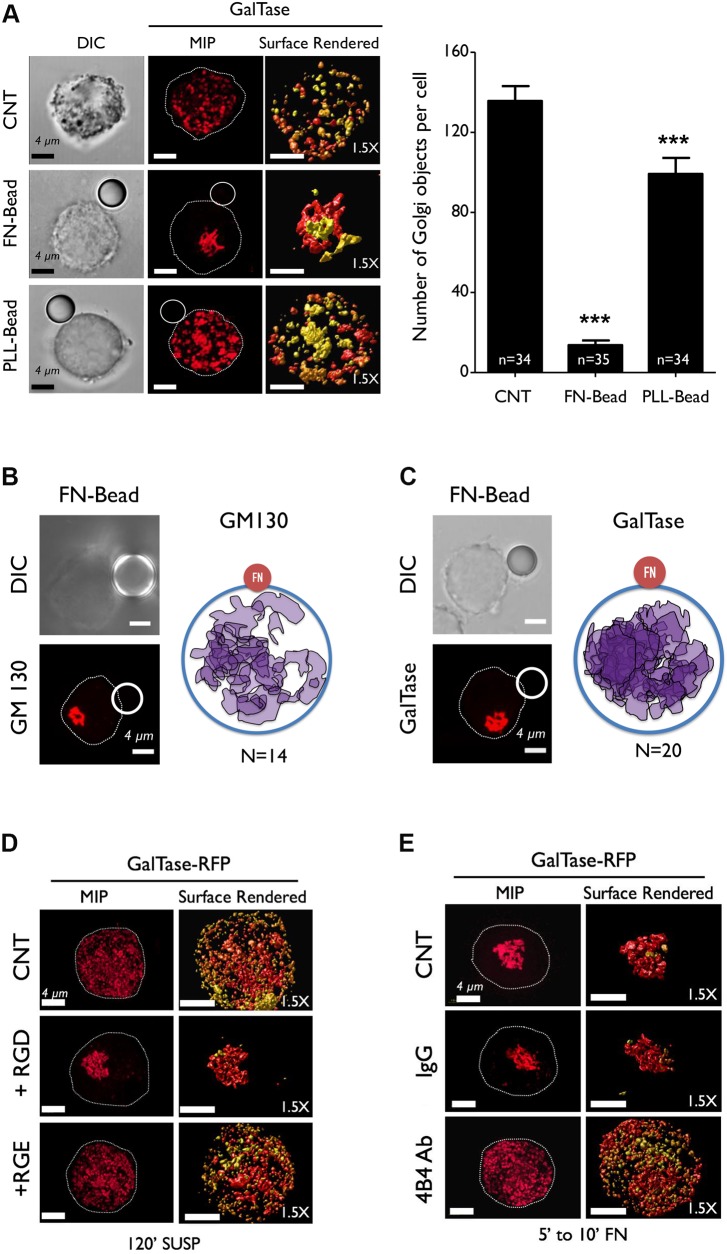
**Integrin-mediated adhesion regulates Golgi organization.** (A) WT-MEFs expressing the trans-Golgi marker GalTase were suspended for 30 min and incubated with uncoated beads (CNT), FN-beads or PLL-beads for 15 min. Representative DIC images of cell and attached bead, MIP and surface rendered de-convoluted *z*-stacks (1.5× magnified) are shown. The graph shows the number of discontinuous trans-Golgi objects per cell as mean±s.e. of 34 cells from 3 independent experiments. (B,C) Representative DIC images and a cross-sectional images of a cell with the attached FN-bead and labeled Golgi (left), and schematic of it (right). The position of the GM130- (B) and GalTase- (C) labeled Golgi was mapped (see Materials and Methods) relative to the FN-bead (FN) for 14 and 20 cells, respectively, (left; from 3 independent experiments) (D) WT-MEFs expressing GalTase (trans-Golgi) and suspended for 120 min (120′ SUSP) were incubated with mock (CNT), RGD peptide (+RGD) or RGE peptide (+RGE) for 15 min. Representative MIP and surface-rendered de-convoluted images (1.5× magnified) of cells are shown. (E) Human fibroblasts (BJ cells) expressing GalTase (trans-Golgi) and suspended for 120 min were incubated with PBS (CNT), anti-mouse (IgG) or β1-integrin function-blocking antibody (4B4 Ab) for 15 min, and re-plated on FN (with respective antibodies) for up to 10 min. Representative MIP and surface-rendered de-convoluted images (1.5× magnified) of cells are shown. Data are representative of 3 independent experiments. Scale bars in all images are 4 µm. Statistical analysis was done using Mann–Whitney U test (***P<0.0001).

The rapid nature of Golgi reorganization depending on cell-matrix adhesion under low serum conditions does suggest a prominent role for integrin-dependent signaling in driving this pathway. In WT-MEFs, integrin-dependent Akt activation (del Pozo et al., 2004) is seen to drop in suspended cells, recovering rapidly upon re-adhesion to FN (Fig. S3A). To evaluate the role of integrins in this pathway we asked whether, in suspended WT-MEFs, the addition of FN-coated beads (FN-beads) known to cluster integrins (Tran et al., 2002) and activate signaling (del Pozo et al., 2004) affects Golgi organization. This was compared to bound poly-L-lysine beads (PLL-beads). The prominent trans-Golgi phenotype (GalTase) (Fig. 1C) was used to evaluate this and other regulatory pathways. Unlike control and PLL-beads (Fig. 3A), FN-beads restored the trans-Golgi organization, which was reflected in a significant drop in the number of their discontinuous objects (see graph in Fig. 3A). PLL-beads caused a small reduction in the number of these objects (see graph in Fig. 3A), not prominent enough to change the trans-Golgi distribution profile (organized versus disorganized) in the cell population (see graph in Fig. S3C). Control cells and cells bound to PLL-beads, hence, show a predominantly disorganized Golgi phenotype – unlike cells bound to FN-beads (Fig. S3C). Relative to cells bound to PLL-beads, those bound to FN-beads also show a modest increase in Akt activation (Fig. S3D), supporting their differential regulation downstream integrin signaling. Like cells bound to FN and PLL beads, suspended cells re-plated on coverslips that had been coated with FN or PLL for 5 min also showed similar differences in the reorganization of the trans-Golgi (GalTase) (Fig. S3B). FN-bead-bound cells also offer a means of evaluating the spatial regulation of adhesion-dependent Golgi reorganization. By using cells bound to a single FN-bead, the localization of the reorganized cis- and trans-Golgi was compared to the position of the bead. The reorganized Golgi did not show any spatial predisposition for the FN bead (Fig. 3B,C) or FN-coated coverslips in re-adherent cells (Fig. S3E). This lack of spatial regulation could also be a reflection of the adhesion-dependent signaling rapidly triggering Golgi reorganization at a pre-determined location, such as around the MTOC (Hurtado et al., 2011).

To further establish the role of integrins, we compared the effect the integrin-binding RGD motif has on Golgi organization in suspended cells with that of an RGE control sequence. RGD peptides – while known to block integrin signaling in adherent cells (Li and Sakaguchi, 2004) – also trigger integrin activation on lipid bilayers, causing the formation of integrin clusters that stimulate actin polymerization (Ye et al., 2012). Addition of soluble RGD peptide to suspended cells is seen to trigger integrin-dependent activation of Src family kinases (Laser et al., 2000; Seong et al., 2013; Zhang et al., 2014). Knowing the effect FN-beads have on Golgi organization, we asked whether addition of RGD (vs RGE) peptide similarly affects the Golgi. Suspended WT-MEFs when treated for an additional 15 min with RGD peptide (Pinkse et al., 2005) showed the trans-Golgi to be reorganized, unlike RGE treated or control cells (Fig. 3D). The distribution profile of the Golgi in this cell population, i.e. suspended WT-MEFs, confirmed RGD-treated cells to have a predominantly organized trans-Golgi phenotype compared with RGE-treated control and untreated cells (Fig. S3F).

Knowing the adhesion-dependent regulation of the Golgi is in response to cells binding to FN, we asked whether targeting β1-integrin (prominently activated upon binding to FN) (Plow et al., 2000) can disrupt Golgi re-organization. Human β1-integrin blocking antibody (4B4 clone) (Arjonen et al., 2012), was used to block its function in human fibroblasts (BJ cells). Serum-deprived BJ cells, detached by using Accutase and suspended for 120 min, were mock treated (CNT), incubated with IgG-mouse control antibody (IgG CNT) or β1-integrin blocking antibody (clone 4B4) for an additional 15 min. When re-plated on FN in the presence of antibody, 4B4-treated cells take longer to attach and spread than those treated with mouse IgG control, failing to reorganize their Golgi (Fig. 3E). The Golgi distribution profile confirmed 4B4-treated cell populations to have a predominantly disorganized trans-Golgi phenotype compared to that of the mouse IgG-treated control cells (Fig. S3G). Together these results confirm that integrin-dependent cell binding to FN mediates Golgi organization. With a known crosstalk between integrin and growth factor in WT-MEFs, we also tested whether the presence of serum growth factors (5% FBS) affects the adhesion-dependent Golgi organization. The cis-medial (ManII) and trans-Golgi (GalTase) rapidly disorganize upon loss of adhesion, and reorganize upon re-adhesion in the presence of serum (Fig. S3H). This suggests integrin-mediated regulation of Golgi organization to not be significantly affected by serum growth factors.

### The microtubule network is essential for adhesion-dependent Golgi organization

**Fig. 4.**
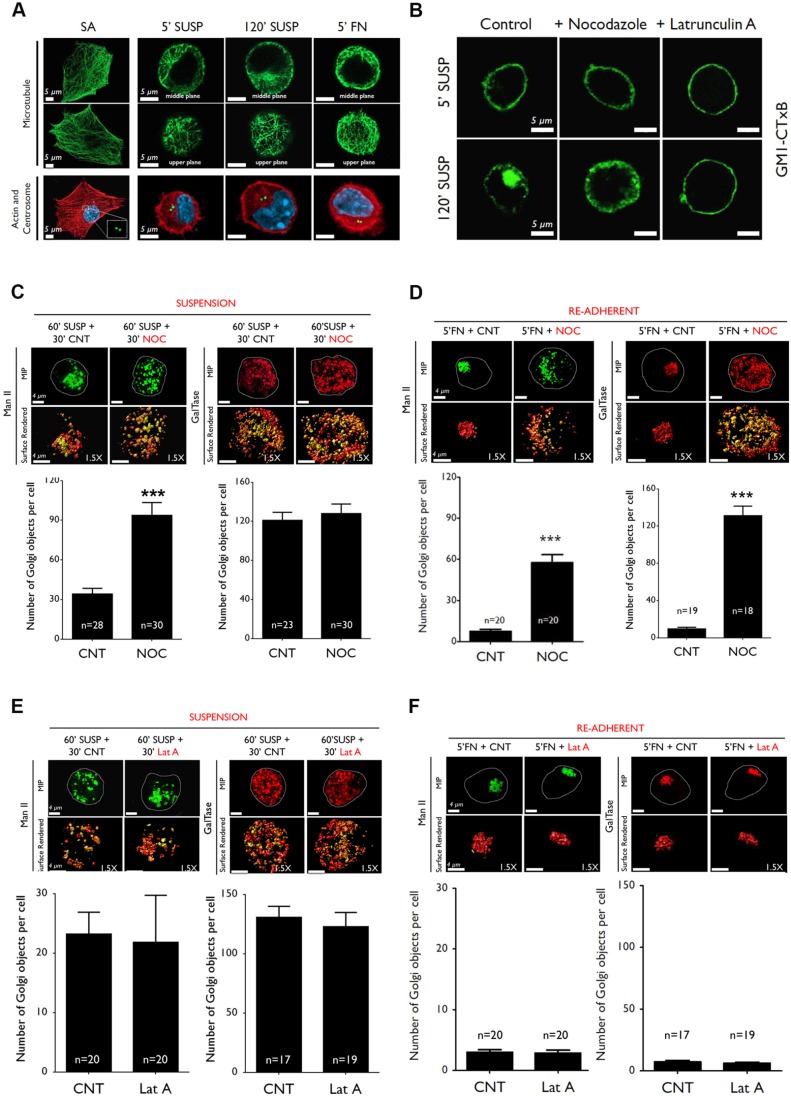
**The role of the cytoskeleton in adhesion-dependent Golgi organization.** (A) Stable adherent (SA), detached (5′ SUSP), suspended (120′SUSP) and re-adherent (5′ FN) WT-MEFs immuno-stained for β-tubulin (microtubules), phalloidin (actin) and γ-tubulin (centrosomes). Representative cross-sectional images of cells stained for microtubules (middle and upper plane of cells) or jointly for actin and γ-tubulin (lower panel) for each time point are shown. Images are representative of 30 cells from 3 independent experiments. (B) WT-MEFs surface labeled with GM1-CTxB were left untreated (control), or were treated with 10 µM Nocodazole (NOC) or 0.5 µM latrunculin A (Lat A), detached (5′ SUSP) and held in suspension for 120 min (120′ SUSP). Representative cross-sectional images from 20 cells (NOC) and 30 cells (Lat A), respectively, from 3 independent experiments. (C–F) WT-MEFs expressing ManII or GalTase were suspended (C,D) for 60 min and additional 30 min not treated (60′SUSP+30′CNT) or treated with NOC (60′SUSP+30′NOC) or Lat A (60′SUSP+30′LatA) and re-plated on FN (E,F) for 5 min without drug (5′FN+CNT) or following treatment with with NOC (5′FN+NOC) (D) or Lat A (5′FN+LatA) (F). Representative MIP- and surface-rendered de-convoluted cells (1.5× magnified) are shown. Graphs show discontinuous cis-medial (ManII) or trans-Golgi (GalTase) objects per cell as mean±s.e. of 18–30 cells (as indicated) from 3 independent experiments. Scale bars: 5 µm (A,B), 4 µm (C–F). Statistical analysis was done using the Mann–Whitney U test (***P<0.001).

Integrin-mediated adhesion is seen to control microtubules (Byron et al., 2015), actin organization (Vicente-Manzanares et al., 2009) and their dynamics (Parsons et al., 2010) to drive cellular polarization, migration and division (Huttenlocher and Horwitz, 2011; Streuli, 2009; Vicente-Manzanares et al., 2009). Both cytoskeletal components are also involved in regulating Golgi organization (Gurel et al., 2014; Zilberman et al., 2011), leading us to ask whether they contribute to the crosstalk between adhesion and Golgi. We first asked whether the cytoskeletal network is functionally compromised upon loss of adhesion. The global microtubule network, stained by using a β-tubulin antibody, in detached, suspended and re-adherent cells is visibly intact (Fig. 4A top two panels), as was the MTOC (stained for γ-tubulin) (Fig. 4A, bottom panel). Whereas prominent actin stress fibers that are seen in stable adherent cells are lost upon loss of adhesion, phalloidin staining of actin did not show a dramatic change in detached versus suspended versus re-adherent cells (Fig. 4A, bottom panel). The functional status of both cytoskeletal networks was evaluated by looking at the effect their disruption, in response to Nocodazole or latrunculin A, has on cytoskeleton-dependent endocytosis of ganglioside GM1 (detected by CTxB labelling of CM1) triggered by loss of adhesion (Balasubramanian et al., 2007b) (Fig. 4B). Treatment with Nocodazole trapped endocytosed GM1 in the cell cortex, blocking its trafficking to the recycling endosome (Fig. 4B, middle panel) (Balasubramanian et al., 2007b). Treatment with latrunculin A blocked GM1 endocytosis at the plasma membrane itself (Fig. 4B, right panel). This shows the actin and microtubule network to be functional upon loss of adhesion, which might contribute to the observed Golgi phenotype.

To test this hypothesis, WT-MEFs suspended for 60 min were treated with Nocodazole (10 µM) for an additional 30 min and the organization of cis-medial (ManII) and trans-Golgi (GalTase) was evaluated. While the trans-Golgi did not show any visible change in organization or number of discontinuous Golgi objects (Fig. 4C, right panel), the cis-medial Golgi was seen to be more disorganized, with a significant increase in the number of discontinuous Golgi objects (Fig. 4C, left panel). Upon re-adhesion, however, both the cis-medial and trans-Golgi failed to reorganize (Fig. 4D). Nocodazole washout from suspended cells followed by their re-adhesion allowed the Golgi to reorganize because the microtubule network had been restored (Fig. S4A). Similarly, disruption of the actin cytoskeleton with latrunculin A (0.5 µM) did not affect the cis-medial or trans-Golgi organization in suspended and re-adherent cells (Fig. 4E,F). Whereas the actin cytoskeleton has been implicated in regulating Golgi organization (Egea and Serra-Peinado, 2014; Liu et al., 2017), surprisingly, it does not contribute to the loss of adhesion phenotype. Integrin-dependent spatial regulation of microtubule organization and dynamics could support adhesion-dependent Golgi organization. A subset of Golgi-derived microtubules are known to be nucleated at the Golgi (Sanders and Kaverina, 2015) and might contribute to such regulation. Microtubule-dependent Golgi assembly is seen to be dependent on the motor protein dynein (Miller et al., 2009), which is recruited by activated Arf1 to Golgi membranes (Yadav et al., 2012). We, hence, asked whether adhesion can regulate Arf1 activation (like Arf6) (Balasubramanian et al., 2007b) and used dynein to control Golgi organization.

### Adhesion-dependent Arf1 activation regulates Golgi organization

**Fig. 5.**
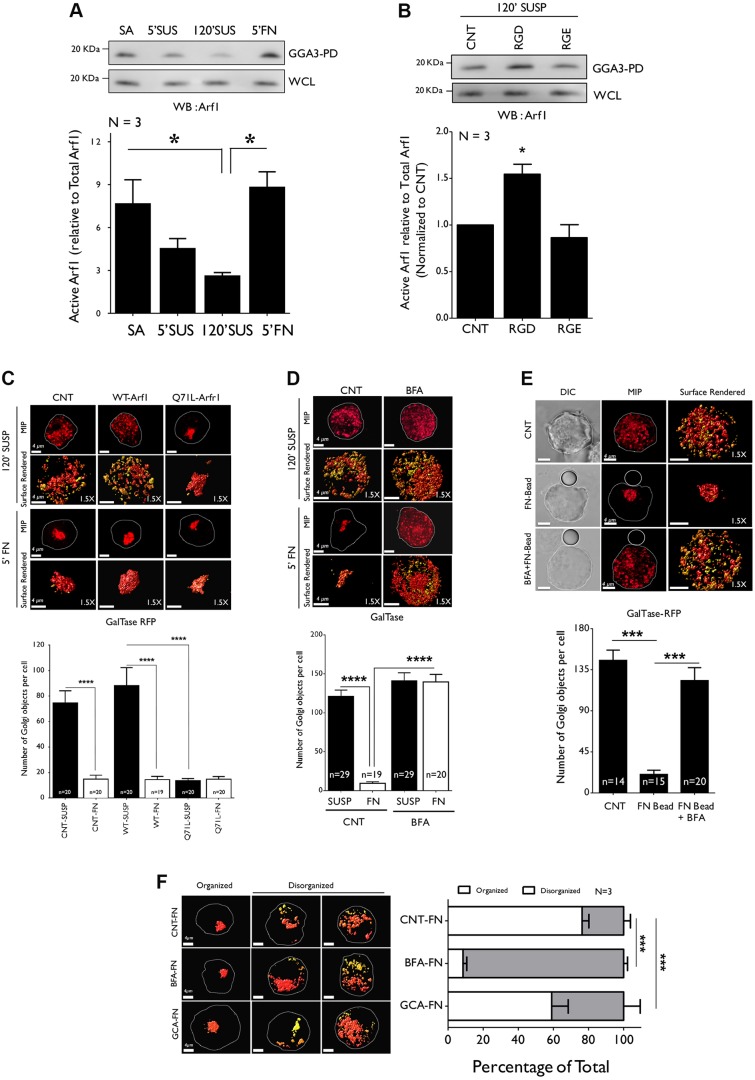
**Adhesion-dependent Arf1 activation regulates Golgi organization.** (A) Western blot detection of activated Arf1 (WB: Arf1) immunostained in GST-GGA3 (GGA3 PD) and total Arf1 in whole-cell lysate (WCL) from stable adherent (SA), detached (5′ SUS), suspended for 120 min (120′ SUS) and re-adherent (5′ FN) WT-MEFs. (B) Western blot of activated Arf1 in GST-GGA3 pulldown (GGA3-PD) and of total Arf1 in WCL of WT-MEFs suspended for 120 min (120′ SUSP) and mock treated with the volume equivalent of Milli-Q water (CNT), or treated with RGD peptide (RGD) or RGE peptide (RGE) in Milli-Q water for 15 min. The graphs in A and B represent the densitometric band intensity ratio of activated Arf1 (GGA3-PD) to total Arf1 as the mean±s.e. from 3 independent experiments. (C) WT-MEFs transfected with GalTase alone (CNT), GFP-tagged WT-Arf1 or constitutively active Q71L Arf1 were suspended for 120 min (120′ SUSP) and re-plated on FN for 5 min (5′ FN). (D) GalTase expressing WT-MEFs suspended for 90 min were treated with methanol as a control (CNT) or (10 µg/ml) BFA for 30 min and re-plated on FN for 5 min (5′ FN) without or with BFA. (E) GalTase-expressing WT-MEFs were suspended for 90 min and incubated for 30 min without beads as control (CNT) or with beads coated with FN and incubated with methanol (FN-Bead) or BFA (BFA+FN-Bead). In all of the above, representative MIP and surface-rendered de-convoluted images (1.5× magnified) of cells are shown, together with DIC images in the FN-bead study. Graphs represent discontinuous Golgi objects per cell as the mean±s.e. of 15–29 cells (as indicated in graph) from 3 independent experiments. (F) WT-MEFs expressing GalTase suspended for 60 min were treated with 10 µg/ml DMSO (mock), 10 µM BFA or 10 µM Golgicide-A (GCA) for 30 min and re-plated on FN alone (CNT-FN) or FN with BFA (BFA-FN) or FN with GCA (GCA-FN). The distribution of cells with organized and disorganized Golgi phenotypes in these populations was determined (in percent) and representative surface-rendered cross-section images are shown. The graph represents mean±s.e. from 3 independent experiments. Scale bars in images is 4 µm. Statistical analysis was done using Mann–Whitney U test and Chi-Square test for distribution profile (*P<0.05, **P<0.001, ***P<0.0001, ns=non-significant).

As a first step in understanding the adhesion-Arf1 connection, we tested activation of Arf1 in stable adherent, detached (5′ SUSP), suspended (120′ SUSP) and re-adherent cells (5′ FN). With no change of total Arf1 levels (Fig. S5A), pulldown of activated Arf1 using the glutathione S-transferase (GST) Golgi-localized γ-ear containing Arf-binding protein 3 (GGA3) fusion protein (GST-GGA3) showed a marginal drop in the numbers of detached cells that decreased significantly upon suspension (∼65% decrease) and was rapidly restored upon re-adhesion to FN (Fig. 5A). This suggests that, like Arf6 (Balasubramanian et al., 2007b), Arf1 activation is regulated by adhesion and might contribute to the observed Golgi phenotype. We further tested the role of integrins by evaluating Arf1 activation in suspended cells incubated with RGD – which is known to restore Golgi organization (Fig. 3D) – compared with suspended cells incubated with RGE. Treatment with RGD peptide significantly increased Arf1 activation relative to RGE control (Fig. 5B) without affecting total Arf1 levels (Fig. S5B). Together, these results suggest that integrin-dependent Arf1 activation contributes to Golgi organization.

To confirm the role of Arf1 activation, we asked whether expression of constitutively active Arf1 (Q71L-Arf1-GFP) (Fig. S5C) in suspended WT-MEFs can bypass the decrease in Arf1 activation in order to reverse Golgi disorganization. We found this was, indeed, the case for the trans-Golgi in cells expressing Q71L-Arf1-GFP, but not those expressing WT-Arf1 (Fig. 5C). Cells expressing constitutively active Arf1 showed a significant decrease in number of discontinuous Golgi objects (see graph in Fig. 5C) and a predominantly organized phenotype in its distribution profile (Fig. S5D). Together these observations confirm that, upon loss of adhesion, the drop in Arf1 activation causes Golgi disorganization, and that this is restored upon re-adhesion-mediated Arf1 activation. It is, however, possible that another adhesion-dependent pathway that is independent of Arf1 contributes to Golgi organization. To test this possibility, we asked whether the Golgi restoration phenotype is affected upon inhibition of Arf1 in re-adherent cells. These inhibition studies are limited by the fact that disrupted Arf1 activation or function are likely to affect Golgi organization (Dascher and Balch, 1994; Fujiwara et al., 1988; Lippincott-Schwartz et al., 1989) independently of adhesion. However, re-adhesion studies might still allow us to comment on the existence of any adhesion-dependent but Arf1-independent regulation of Golgi organization in these cells.

We, therefore, treated suspended cells that carried a disorganized Golgi with the known Arf1 inhibitor BFA (Donaldson et al., 1992) and found that treatment with BFA affected the cis-medial Golgi (Fig. S2A), causing it to fall back into the ER; the trans-Golgi organization in these cells, however, was largely unaffected (Fig. 5D). Re-adhesion of BFA-treated cells, unlike mock-treated control cells, failed to restore Golgi integrity (Fig. 5D). BFA-treated cells in suspension that were incubated with FN-beads (BFA+FN Bead) also did not show a rescue of the disorganized Golgi phenotype (Fig. 5E). This was reflected in the number of trans-Golgi objects staying significantly high in BFA-treated re-adherent cells (see graph in Fig. 5D) and BFA treated cells bound to FN-beads, relative to their respective controls (see graph in Fig. 5E). This was further confirmed by the predominantly disorganized trans-Golgi phenotype in both cell populations (Fig. S5E,F). Inhibition of Arf1 activation with a dominant-negative Arf1 mutant (GFP tagged T31N Arf1) (Dascher and Balch, 1994) (Fig. S5C) also did not significantly affect the trans-Golgi phenotype (GalTase) in suspended cells (Fig. S5G). When re-plated on FN, T31N-Arf1-expressing cells retained their disorganized trans-Golgi phenotype relative to untreated control (Fig. S5G). This was also reflected in the number of trans-Golgi objects staying significantly high (see graph in Fig. S5G). Together these results suggest adhesion-dependent Arf1 activation to be a main player, mediating Golgi organization in these cells.

BFA acts as a non-competitive inhibitor of Arf GEFs, the BFA-inhibited GEFs BIG1 and 2, and Golgi-specific BFA-resistance factor 1 (GBF1) (Lowery et al., 2013). In contrast, Golgicide-A (GCA) acts only on GBF1 (Sáenz et al., 2009). We, hence, used the above described assay to treat suspended cells with GCA inhibitor and re-plate them on FN to test the relative effect BFA and GCA have upon re-adhesion-mediated Golgi re-organization. Whereas BFA treatment blocked Golgi reorganization dramatically (∼92% of cells remain disorganized) GCA had a lesser effect (∼41% cells remain disorganized). Both, however, prevented Golgi reorganization significantly better than control cells (∼24% of cells remain disorganized) (Fig. 5F). This showed that treatment with GCA was able to inhibit adhesion-dependent Golgi organization but significantly less than BFA. This suggests a more prominent role for BIG1/2 (over GBF1) in the adhesion-dependent Golgi organization. siRNA knockdown studies have suggested that Golgi-localized Arfs 1, 3, 4 and 5 can, at times, be functionally redundant (Volpicelli-Daley et al., 2005). It is, hence, possible that the prominent effect for BIG1/2 is in part mediated by its regulation of multiple Golgi Arf(s) (D'Souza-Schorey and Chavrier, 2006). The role of Arf proteins other than Arf1 might have in adhesion-dependent Golgi organization does, however, need careful evaluation.

### Activated Arf1 binds dynein to regulate Golgi organization following adhesion

**Fig. 6.**
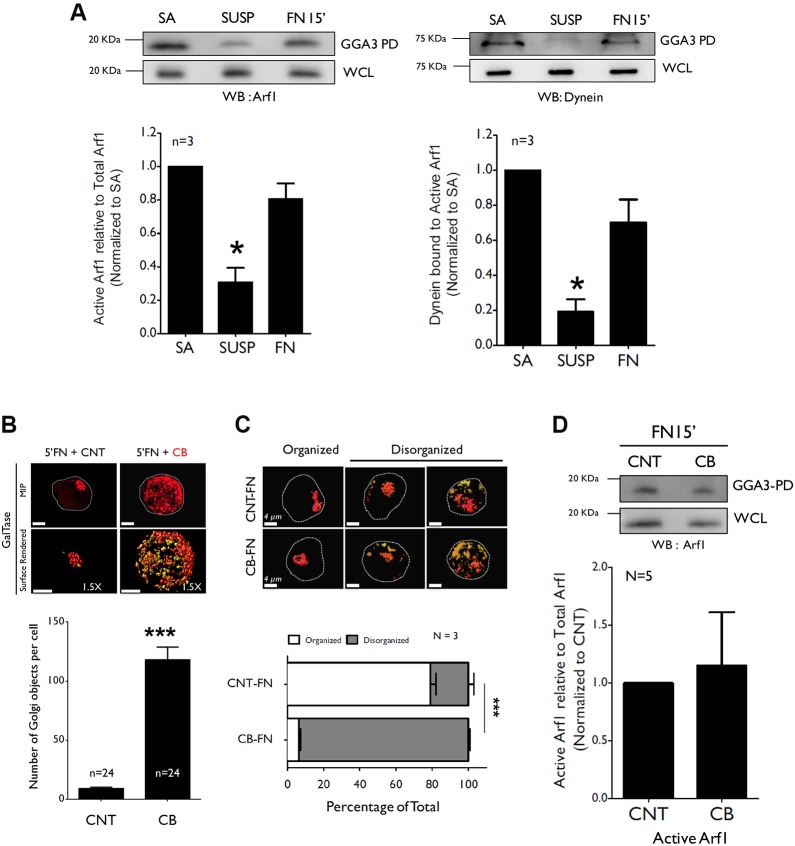
**Association of activated Arf1 with dynein regulates Golgi organization.** (A) Western blot of activated Arf1 (WB: Arf1) and dynein (WB: Dynein) pulled down with GST-GGA3 (GGA3 PD) and total Arf1 and dynein in whole-cell lysate (WCL) from stable adherent (SA), suspended (120′ SUS) and re-adherent (15′ FN) WT-MEFs. Ratio of densitometric band intensities of Arf1 and dynein in GGA3-PD relative to their levels in the WCL are represented in the graph as mean±s.e. from 3 independent experiments. (B) WT-MEFs expressing GalTase were held in suspension for 90 mins were treated with DMSO or 20 µM Ciliobrevin-D (CB) for 30′ and re-plated on FN without (5′FN+CNT) or with CB (5′FN+CB). Representative MIP and surface rendered de-convoluted images (1.5× magnified) of cells are shown. Graph represents discontinuous Golgi objects per cell as mean±s.e. from 24 cells from 3 independent experiments. (C) Percentage distribution of WTMEFs with organized and disorganized Golgi phenotypes in re-adherent control (CNT-FN) or Ciliobrevin (CB-FN) treated cells. Representative surface rendered cross-section images shown. The graph represents mean±s.e. from 3 independent experiments. (D) Western blot detection of activated Arf1 (WB: Arf1) pulled down with GST-GGA3 (GGA3 PD) and total Arf1 in WCL from WT-MEFs re-adherent on FN (15′FN) WT-MEFs without (CNT) or with Ciliobrevin (CB). Ratio of densitometric band intensities of Arf1 in GGA3-PD relative to their levels in the WCL are represented in the graph as mean±s.e. from 5 independent experiments. Scale bars: 4 µm. Statistical analysis was done using one sample *t*-test except for the distribution profiles where χ^2^-test was performed (**P<0.001, ***P<0.0001).

With activated Arf1 preferentially binding the Golgi (Donaldson et al., 2005) adhesion-dependent activation of Arf1 could control its localization at the Golgi membrane, in turn, mediating the recruitment of minus-end motor protein dynein to control Golgi organization (Yadav et al., 2012) along the microtubule network (Fig. 4C,D). We, indeed, found that pulldown of activated Arf1 with GST-GGA3 brings down dynein with it, whereas control GST-Sec5 Ral-binding domain (GST-Sec5 RBD) pulldown (Pawar et al., 2016) did not bind Arf1 or dynein (Fig. S6A). Inhibition of Arf1 with BFA reduces activated Arf1 pulled down with GST-GGA3, and dynein brought down with it (Fig. S6B). Binding of dynein to activated Arf1 pulled down with GST-GGA3 but not GST-Sec5 RBD supports their association to, indeed, be specific. Levels of activated Arf1 in GST-GGA3 pulldowns dropped significantly in suspended cells (120′ SUSP) (Fig. 6A, left panel), together with a comparable reduction in the amount of dynein brought down with it (Fig. 6A, right panel). Re-adhesion of cells on FN for 15 min (FN 15′) restored Arf1 activation and dynein bound in GST-GGA3 pulldowns (Fig. 6A). We also looked at GST-GGA3 pulldown assays of stable adherent, detached (5′ SUSP), suspended (120′ SUSP) and cells re-adherent on FN for 5 min (5′ FN) and found a comparable change in activated Arf1 pulled down and dynein brought down with it (Fig. S6C). This suggests that, upon loss of adhesion, a drop in activated Arf1 levels and, hence, dynein bound with it affect their levels on the Golgi, allowing it to now disorganize along the microtubule network. Re-adhesion restores Arf1 activation and dynein recruitment in order to restore the organized Golgi phenotype. To confirm this, we treated suspended cells (with a disorganized Golgi) with the hedgehog pathway inhibitor Ciliobrevin D to block dynein function and found that reorganization of the trans-Golgi (GalTase) was blocked upon re-adhesion to FN for 5 min (5′ FN) (Fig. 6B). This was reflected in the increased number of discontinuous Golgi objects (see graph in Fig. 6B) and a predominantly disorganized Golgi phenotype seen in ciliobrevin-treated cells (Fig. 6C). Ciliobrevin did not affect adhesion-dependent Arf1 activation (relative to untreated control) (Fig. 6D) or net Arf1 levels (Fig. S6D), suggesting re-adhesion-mediated activation of Arf1 – although normal in ciliobrevin-treated cells – cannot support Golgi reorganization in the absence of functional dynein. Together these findings reveal the presence of an integrin–activated Arf1–dynein–microtubule pathway that controls adhesion-dependent Golgi organization.

### Adhesion-dependent Golgi organization affects Golgi function

**Fig. 7.**
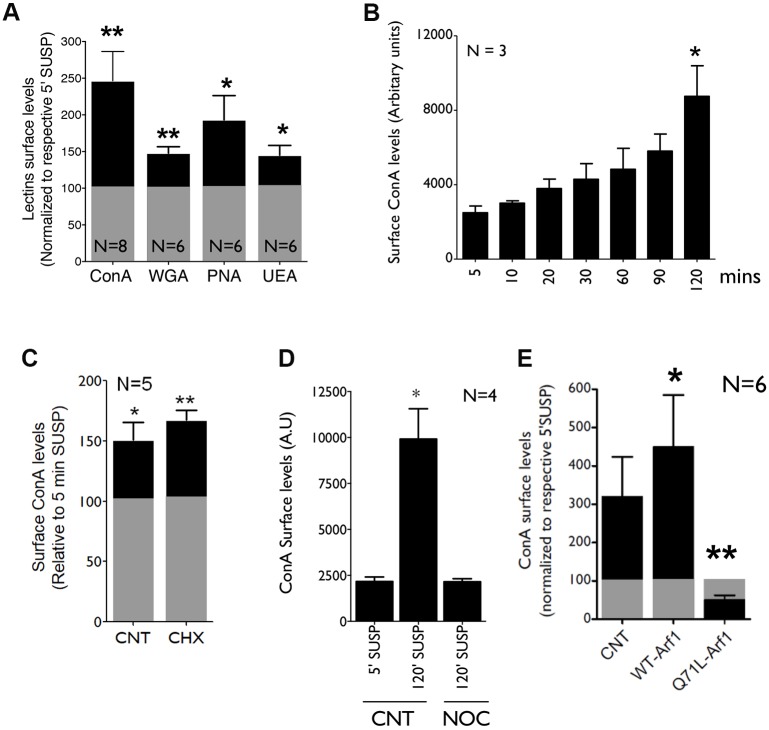
**Loss of adhesion mediated Golgi disorganization affects Golgi function.** (A) WT-MEFs detached (5′ SUSP) with Accutase and held in suspension for 120 min (120′ SUSP) were labeled with ConA-Alexa 488, WGA, PNA and FITC-UEA lectin. Median fluorescence of cell surface-bound lectin fluorescence measured by flow cytometry at 120′ SUSP (black bars) was normalized to levels at 5′ SUSP (grey bars). The graph represents mean±s.e. from 8 (ConA) and 6 (WGA, PNA, UEA) independent experiments. (B) WT-MEFs detached (5′) and suspended for 10, 20, 30, 60, 90 and 120 mins and labeled with ConA-Alexa 488. Graph shows median fluorescence intensity as mean±s.e. from 3 independent experiments. (C) Cells untreated (CNT) or treated with 20 µg/ml CHX for 4 h were detached (5′ SUSP), held in suspension for 120 min (120′ SUSP) and labeled with ConA-Alexa 488. Median fluorescence measured by flow cytometry in 120′ SUSP (black bars) were normalized to levels in 5′ SUSP (grey bars) and are represented in the graph (mean±s.e.) from 5 independent experiments. (D) Detached WT-MEFs (5′ SUSP), suspended for 90 min and treated with DMSO (CNT) or Nocodazole (NOC) for 30 min were labeled with ConA-Alexa 488. Median fluorescence intensity is represented in the graph (mean±s.e.) from 4 independent experiments. (E) WT-MEFs expressing mCherry-N1 (CNT), WT-Arf1-mCherry (WT-Arf1) or Q71L-Arf1-mCherry (Q71L-Arf1) were labeled with ConA-Alexa 488. Median lectin fluorescence intensity in cell population gated for Arf1 expression was measured and median fluorescence intensity in 120′ SUSP cells (black bars) and normalized intensity in cells when detached (5′ SUSP cells; grey bar). The graph represents mean±s.e. of 6 independent experiments. Statistical analysis was done using Mann–Whitney U (B,D) and one sample *t*-test (A,C,E); *P<0.01, **P<0.001, ****P<0.00001.

It is of much interest to test whether and how changes in Golgi organization upon loss of adhesion and re-adhesion affect Golgi function. One major read-out of Golgi function in cells is their ability to glycosylate and deliver proteins and lipids at the plasma membrane. Both N- and O-glycosylation involve a series of enzymatic reactions catalyzed by glycan-processing enzymes across the cis, medial and trans-Golgi compartments (Stanley, 2011; Varki, 1998). Changes in Golgi organization does affect processing and trafficking of glycosylated proteins and lipids (Pokrovskaya et al., 2011), which can be detected using lectins that selectively recognize glycan epitopes (Sharon and Lis, 2004). Using flow cytometry, we quantitated how loss of adhesion affects the cell membrane binding (and hence levels) of fluorescently tagged lectins, concanavalin A (ConA; i.e. mannose-binding), wheat germ agglutinin (WGA; i.e. galactose/N-acetylgalactosamine binding), peanut agglutinin (PNA; i.e. N-acetylglucosamine binding) and *Ulex europaeus* agglutinin (UEA; i.e. fucose binding). Levels of surface-bound lectin in detached cells (5′ SUSP) when normalized to control (100, grey bars) show relative levels in suspended cells (120′ SUSP) to be significantly increased for WGA, PNA, UEA and ConA (black bars) (Fig. 7A). ConA-bound surface lectin levels showed the most change upon loss of adhesion and were used to further evaluate the regulation of this pathway. We first tested the kinetics of ConA-lectin binding upon loss of adhesion using cells suspended for 5, 10, 20, 30, 60, 90 and 120 min (Fig. 7B). This revealed the increase in cell surface glycosylation (detected by ConA binding) to be gradual, with a significant change detected at 120 min suspension (Fig. 7B). This could reflect a change in the rate at which glycosylated proteins are synthesized, processed and/or delivered from the Golgi to the plasma membrane. To test whether new protein synthesis contributes to this increase, we pre-treated cells with cycloheximide (CHX) to block protein synthesis and evaluated the change in surface ConA binding. CHX treatment did not affect the increase in surface ConA binding upon loss of adhesion (Fig. 7C), suggesting protein synthesis to not be a contributing factor to this increase. Knowing the role microtubules have in regulating Golgi organization (Fig. 4C,D) and trafficking (Fig. 4B), we pre-treated suspended cells with Nocodazole to ask whether and how it affects the change in cell surface glycosylation (ConA binding). Nocodazole treatment was seen to enhance Golgi disorganization in suspended cells (Fig. 4D) but blocked the increase in cell surface ConA-lectin binding (Fig. 7D). This suggests that microtubule-dependent trafficking supports changes in cell surface glycosylation upon loss of adhesion. It also implies that the disorganized nature of the Golgi upon loss of adhesion – if further disrupted – does not support the change in cell surface glycosylation.

**Fig. 8.**
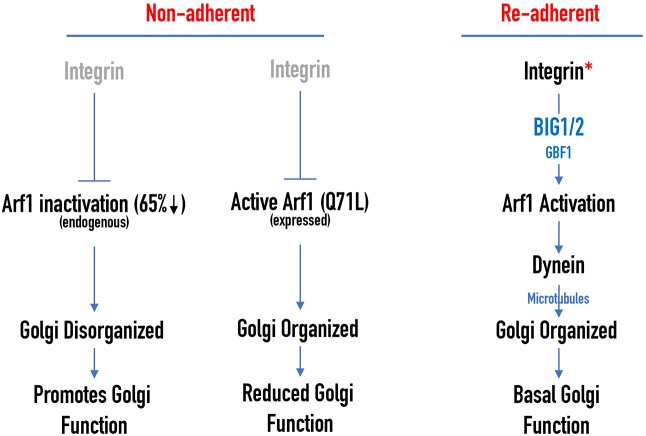
**Proposed model for adhesion-dependent Golgi organization.** Schematic shows integrin-mediated adhesion prominently uses Arf1 GEF BIG1/2 (over GBF1) to activate Arf1, which recruits the microtubule motor protein dynein to control Golgi organization and function.

To confirm whether, indeed, the loss of adhesion-mediated change in glycosylation is caused by the disorganized Golgi phenotype, we used active Arf1 (Q71L) to restore Golgi integrity in suspended cells (Fig. 5C) and asked whether and how this affected cell surface glycosylation levels. Detached (5′ SUSP) WT-Arf1- and active Q71L-Arf1-expressing cells show comparable Arf1 expression (Fig. S7A) and a modest change in basal cell surface ConA-lectin binding (Fig. S7B). When held in suspension (120′ SUSP) active Q71LArf1-expressing cells did not show an increase in surface ConA binding, as seen in WT Arf1 and control (CNT) cells (Fig. 7E). This suggests that active Arf1-mediated restoration of Golgi integrity (Fig. 5C) prevented the increased cell surface glycosylation observed in suspended cells (Fig. 7E). Together, these results confirm integrin-mediated adhesion and its regulation of Arf1, to control Golgi organization and function (Fig. 8).

## DISCUSSION

The adhesion-dependent regulation of Golgi organization and function might contribute to the many signaling pathways and processes that control cell binding to the extracellular matrix. These include cell polarization, migration, division and anchorage-dependent signaling (Ben-Ze'ev and Raz, 1981; Cox et al., 2001; Huttenlocher and Horwitz, 2011; Schwartz and Assoian, 2001). A role for β1-integrins in the regulation of adhesion-dependent Golgi organization had been revealed in these earlier studies, although how this and other integrins drive this pathway remains to be fully understood. Limited clustering and/or activation of integrins (as seen on FN-bead- or RGD-bound cells) can activate Arf1 to drive Golgi re-organization. The rapid nature of this integrin-dependent response supports a downstream role for a swiftly triggered and disseminated regulator, such as Ca^2+^. Other studies have shown that integrin activation drives transient changes in intracellular Ca^2+^ levels (Balasubramanian et al., 2007a; Wu et al., 2001). Ca^2+^-dependent interaction between the neuronal Ca^2+^ sensor NCS-1 and Arf1 on the Golgi has been found to control its organization (Haynes et al., 2005). While our study here has commented on the role of Arf1 GEFs downstream of adhesion, a role for Arf GAPs in regulating this pathway is just as likely. Interestingly, Ca^2+^ was found to stimulate the activity of the Arf GAP ASAP3, known to localize at focal adhesion and the Golgi (Ha et al., 2008). Members of the ASAP family of GAPs are thought to be important mediators of the crosstalk between Ca^+2^ and Arf signaling, and might be equally relevant in the integrin–Arf1–Golgi pathway (Ismail et al., 2010). With significant overlap in GEFs and GAPs that regulate Arfs and several Arfs localizing at the Golgi (D'Souza-Schorey and Chavrier, 2006), and at least two (Arf6 and – now – Arf1) known to be regulated by adhesion (Balasubramanian et al., 2007b), additional Golgi-associated Arfs might also contribute to this crosstalk. Integrins at the plasma membrane do regulate microtubule dynamics (Byron et al., 2015; Wickström et al., 2010), and might similarly affect Golgi associated microtubules (Sanders and Kaverina, 2015) to influence Golgi organization.

Also, unique to this regulation of the Golgi by adhesion is the differential effect loss of adhesion has on cis/cis-medial versus trans-Golgi/TGN. Although the differential composition, role and regulation of individual Golgi compartments is known (Papanikou and Glick, 2014; Sens and Rao, 2013), their spatial separation in suspended cells could provide an attractive tool to evaluate their crosstalk. Previous studies have suggested that the Arf1 GEFs BIG1 and BIG2 preferentially localize to the trans-Golgi, whereas GBF1 prefers the cis-compartment (Manolea et al., 2008). In our current study BIG1 and 2 (more than GBF1) were seen to regulate adhesion-dependent Golgi reorganization. Together, this could explain the trans-Golgi being consistently more disorganized than the cis and cis-medial Golgi upon loss of adhesion. While net Arf1 activation upon the loss of adhesion decreases by ∼65%, its effect on local Arf1 activation in the trans versus cis/cis-medial Golgi might, indeed, be variable. This could then differentially affect dynein recruitment and disorganization of cis versus trans-Golgi (Yadav et al., 2012). While our current study reveals a role for the minus-end motor protein dynein in mediating adhesion-dependent Golgi re-organization (through activated Arf1), the possible role plus-end kinesin motor proteins have in this pathway should not be ignored. The kinesin-like protein KIF1C binds the Golgi protein Rab6A to regulate Golgi fragmentation (Lee et al., 2015). The levels of this and other kinesin motors, relative to that of bound dynein, could affect directional movement of Golgi membranes on the microtubule network. Downstream of adhesion processes such the dynein–kinesin tug-of-war could drive relative Golgi disorganization.

Upon loss of adhesion, the disorganized Golgi phenotype is distinctly different from BFA-mediated Golgi fragmentation that causes the cis-Golgi to retreat into the ER (Fujiwara et al., 1988; Lippincott-Schwartz et al., 1989). In non-adherent cells, the disorganized cis-Golgi shows almost no overlap with the ER until these cells are treated with BFA. Compared with stable adherent cells, non-adherent cells show ∼65% drop in levels of activated Arf1 that, upon treatment with BFA, drops further to ∼86%. This raises the possibility that changing levels of activated Arf1 progressively affect Golgi organization. Upon loss of adhesion, the resulting disorganized Golgi could, hence, be an intermediate to Golgi fragmentation. Golgi fragmentation and partitioning is necessary for cells to progress through mitosis (Corda et al., 2012) and is regulated by Arf1 (Altan-Bonnet et al., 2003). Integrin-mediated adhesion is known to control cell cycle progression, helping to determine spindle orientation in a dividing cell (Lancaster et al., 2013). A dividing cell is seen to undergo ‘mitotic cell rounding’ driven by detachment and an increase in RhoA-dependent cell cortex retraction (Maddox and Burridge, 2003). Regarding mitotic cell rounding, it remains unclear, whether changes in cell adhesion could affect Golgi organization, eventually supporting Golgi fragmentation.

A change in Golgi function might reflect altered glycosylation, trafficking and sorting (Xiang et al., 2013). Accurate glycosylation is essential for cell adhesion, migration and cell–cell communication (Ohtsubo and Marth, 2006). Glycosylation of cell surface receptors affects their conformation (Takahashi et al., 2009), ligand binding, dimerization capability (Isaji et al., 2009) and downstream signaling (Zhao et al., 2008b). In polarized epithelial cells glycosylation is also seen to control spatial targeting of proteins (Weisz and Rodriguez-Boulan, 2009), making its regulation vital. Integrins themselves are glycosylated, affecting their ability to form functional dimers that, in turn, control their activation during cell adhesion (Gu et al., 2012) and migration (Janik et al., 2010). Glycosylation of β1-integrin is regulated by BIG1-dependent Arf1 activation (Shen et al., 2007). Loss of adhesion-mediated Golgi disorganization could, hence, affect β1- and other integrin glycosylation and function. Cell–cell interactions through E-cadherins can also be regulated by glycosylation that affects their intercellular binding kinetics and adherent junction formation (Langer et al., 2012; Zhao et al., 2008a). Loss of adhesion-mediated regulation of the Golgi and, particularly, the TGN could also affect Golgi-dependent trafficking and sorting (Guo et al., 2014) that, in part, contributes to observed changes in glycosylation at the cell surface.

Integrin-dependent adhesion regulates membrane trafficking to control anchorage-dependent signaling, that is deregulated in cancer (Balasubramanian et al., 2007b, 2010; Pawar et al., 2016; Wickström and Fässler, 2011). Changes in Golgi organization and function might contribute to how proteins and lipids are processed and delivered (Bankaitis et al., 2012; Rodriguez-Boulan and Müsch, 2005), which could also affect anchorage-dependent signaling. Malignant transformation in cancer cells is accompanied by aberrant glycosylation of proteins, including integrins and cadherins (Kariya et al., 2017; Varki et al., 2009). In many cancers, the Golgi is fragmented to drive this change (Migita and Inoue, 2012; Petrosyan, 2015). It will be important to investigate whether and how changes in surface glycosylation upon loss of adhesion compare to those seen upon oncogenic transformation and their relative contributions to anchorage-independent signaling. By identifying integrin-dependent cell-matrix adhesion as a regulator of Golgi organization and function, our study highlights the role such a regulatory pathway might have under normal and pathological conditions.

## MATERIALS AND METHODS

### Reagents

Fibronectin (FN) was purchased from Sigma (cat. no. F2006). Cholera toxin subunit B (CTxB) conjugated with Alexa Fluor 594 (C22843) or Alexa Fluor 488 (C34775) were purchased from Invitrogen Molecular Probes. Accutase was purchased from Sigma (cat. no. A6964). Lectin probes, concanavalin A conjugated to Alexa Fluor 488 (ConA-Alexa 488; cat. no. C11252), PNA-Alexa Fluor 488 (cat. no. L21409) and WGA-Alexa Fluor 488 (cat. no. W11261) were purchased from Invitrogen Molecular Probes. UEA-FITC was purchased from Sigma (cat. no. L9006). Divinyl polystyrene beads (cat. no. 42045A1) were purchased from Thermo Scientific. Nocodazole (cat. no. M1404), latrunculin A (cat. no. L5163), brefeldin A (cat. no. B7651), Golgicide A (cat. no. G0923), cycloheximide (CHX, cat. no. C6255), were purchased from Sigma. RGD peptide (cat. no. 4027371) and RGE peptide (cat. no. 4040481) were from Bachem. Ciliobrevin D (cat. no. 250401) was purchased from Calbiochem. Fluoromount-G (cat. no. 0100-01) was purchased from Southern Biotech.

### Antibodies

For western blots, the following antibodies were used: anti-Arf1 (clone 1D9, Abcam, cat. no. ab2806) at a dilution of 1:500, anti-Arf1 (clone EP442Y, abcam, cat. no. ab32524) at a dilution of 1:500, anti-dynein (clone 74.1, Millipore, cat. no. MAB1618) at a dilution of 1:2000, anti-GFP [Santa Cruz, cat. no. GFP (FL): sc-8334] at a dilution of 1:700, anti- β-tubulin (clone E7, Developmental Studies Hybridoma Bank, cat. no. AB_2315513) at a dilution of 1:5000, anti-HA.11 epitope tag antibody (Clone 16B12, Covance, cat. no. MMS-101R) at a dilution of 1:2000. Secondary antibodies conjugated to horseradish peroxidase (HRP) were purchased from Jackson ImmunoResearch, and used at a dilution of 1:10,000 unless specified otherwise.

For immunofluorescence, the following antibodies were used: anti-GM130 (BD Transduction, clone 35, cat. no. 610822) at a dilution of 1:100, mouse anti-p115 (BD Transduction, cat. no. 612260) at a dilution of 1:100, anti-syntaxin-6 (BD Transduction, cat. no. 610635) at a dilution of 1:100, anti-β1-integrin (clone 4B4, cat. no. 6603113, Beckman Coulter) (10 µg/ml), anti-β-tubulin (Clone E7, DHSB, cat. no. AB_2315513) at a dilution of 1:1000, anti-γ-tubulin antibody (Abcam, cat. no. ab11317) at a dilution of 1:100, anti-HA antibody (Clone 3F10, Roche, cat. no. 11867423001) at a dilution of 1:1000. Alexa Fluor 594 conjugated to phalloidin (Invitrogen, cat. no. A12381) was used at a dilution of 1:100.

Secondary antibodies conjugated to Alexa Fluor 488 or Alexa Fluor 594 were purchased from Invitrogen Molecular Probes (cat. no. A12379 and A12381) and used at a dilution of 1:1000 (unless otherwise specified). Goat anti-mouse IgG Antibody, Fc (cat. no. AQ127) was purchased from Millipore and used at 10 µg/ml.

### Plasmids

GFP-tagged Arf1-WT and Arf1-T31N constructs were obtained from Dr Satyajit Mayor (National Centre for Biological Sciences, Bangalore, India). GFP-tagged Arf1-Q71L construct was made by site-directed mutagenesis using GFP-Arf1-WT as the template and following primers - (forward) 5′-GACGTGGGTGGCCTGGACAAGATCCGG-3′ and (reverse) 5′-CCGGATCTTGTCCAGGCCACCCACGTC-3′. mCherry-tagged Arf1-WT and Arf1-Q71L constructs were made by releasing the Arf1 gene from GFP constructs (using Bgl II and BamH1 sites) and cloning the same into an empty mCherry-N1 vector. GalTase-RFP, mannosidase II-GFP, and KDEL-RFP and TGN38-RFP constructs were all obtained from Dr Jennifer Lippincott-Schwartz (NIH). All of the above-mentioned constructs were sequenced to confirm their identity before being used in our experiments.

### Cell culture and transfections

Mouse embryonic fibroblasts (WT-MEFs) obtained from Dr Richard Anderson (University of Texas Health Science Center, Dallas, TX) were cultured in complete Dulbecco's modified Eagle's medium (DMEM) (Invitrogen) with 5% fetal bovine serum (FBS) and penicillin-streptomycin (Pen-Strep; Invitrogen) at 37°C in a 5% CO_2_ incubator**.** Human foreskin fibroblasts (BJ) cells from ATCC (ATCC CRL-2522) were cultured in complete DMEM with 10% FBS and Pen-Strep at 37°C in a 5% CO_2_ incubator**.** All cell lines used were routinely tested for mycoplasma contamination. Cells were transfected using LTX-PLUS (Invitrogen) according to the manufacturer's protocol. Transfections were done in 6-well plates or 6-cm dishes with complete medium using 2 µg or 4 µg DNA, respectively, for 12 h (for all constructs used). At 36 h after transfection, cells were serum deprived for 12 h in low-serum DMEM (containing 0.2% FBS) and then used for experiments. When cells were not serum starved, they were allowed to grow in medium supplemented with 5% FBS for 12 h and then used for experiments.

### Suspension and re-adhesion of cells

WT-MEFS or Human fibroblasts (BJ cells) were cultured in their respective growth medium in 6 cm dishes to ∼75% confluence. Cells were serum deprived for 12 h by culturing them in low-serum DMEM, detached using trypsin-EDTA (Invitrogen) or Accutase (Sigma) (when specified) at 37°C and washed with low-serum DMEM; one aliquot of cells (4×10^5^ cells) was used per time point. This processing took ∼5 min and these detached cells are, therefore, referred to as 5′ SUSP cells. The remaining cells were held at room temperature (RT), added to 20 ml of low-serum DMEM, gently mixed with equal volume of 2% methylcellulose in low-serum DMEM and incubated at 37°C for 120 min (120′ SUSP cells). Following incubation, cells were collected at the required time, carefully washed twice with low-serum DMEM and centrifuged at 367 ***g*** for 5 min at 4°C. They were then reconstituted in low-serum DMEM and re-plated on coverslips coated with 2 µg/ml FN for 5 min (referred to as 5′ FN cells). Cells re-plated on FN were allowed to stay adherent for 4 h and defined as being stable adherent. Coverslips were coated with FN overnight at 4°C, washed with PBS twice and incubated with low-serum DMEM at 37°C for 60 min before cells were plated on them. For confocal microscopy, suspended or re-adherent cells were fixed with 3.5% paraformaldehyde (PFA) for 15 min at RT, washed with PBS thrice, stained and mounted using Fluoramount-G. For western blotting, suspended and re-adherent cells were lysed in 1× Laemmli buffer, heated at 95°C for 5 min and stored at −80°C.

### Immunofluorescence staining for Golgi markers GM130, p115 and syntaxin-6

Serum-deprived cells held in suspension or cells that had re-adhered on FN were fixed with 3.5% PFA at RT, and permeabilized with PBS containing 5% BSA and 0.05% Triton X-100 for 10 min at RT. Cells were then blocked with 5% BSA in PBS for 30 min at RT and incubated with anti GM130 or p115 or syntaxin-6 antibody diluted 1:100 in PBS with 5% BSA for 1 h at RT. Cells were washed with PBS and incubated with 1:1000 diluted anti-mouse Alexa Fluor 488 or Alexa Fluor 568 antibody (as required) at RT for 1 h. Suspended cells were similarly labeled in Eppendorf tubes where the reagent volume was always 50 µl (for blocking, permeabilization, primary antibody and secondary antibody) and cells were prevented from settling by regular tapping of the tube. Cells were washed with twice with 1×PBS and eventually reconstituted in 10 µl PBS, mounted using Fluoromount-G, allowed to dry for 24 h and imaged using a confocal microscope.

### Confocal microscopy

Cells were imaged by using a Zeiss 710 or 780 laser scanning confocal microscope with a 63× oil objective (NA 1.4). Acquisition settings were kept constant, with laser power=2%, Pinhole=1 AU, gain=700–800. Images were acquired at a resolution of 1024×1024. *Z*-stacks were acquired at 0.2 µm intervals, de-convoluted and rendered.

### De-convolution of *z*-stacks and object analysis

All images were processed and analyzed by using the Huygens Professional version 16.10 (Scientific Volume Imaging, The Netherlands, http://svi.nl). De-convolution of confocal *z*-stacks was optimized using the following settings: average background value =1, number of iterations =30, signal to noise ratio (SNR) =20, quality change threshold =0.0001. These settings were kept constant for all image de-convolutions. Point spread function (PSF) values were estimated for each *z*-stack and provide the minimal voxel size the confocal microscope could resolve. This PSF value was then used in the software as garbage volume for surface rendering and object analysis. De-convoluted images were rendered either as a 3D maximum-intensity projection (MIP; by using the MIP renderer plug-in) or surface rendered (using the Surface render plug-in with a 15% primary threshold). Surface rendered images were pseudocolored using the software to distinctly mark discontinuous objects. The top view of a MIP or surface-rendered cell (magnified 1.5× or 2.5×) was used to represent the Golgi phenotype. When needed, a cross-sectional view along the *z*-axis of the MIP image was used to observe the localization of the Golgi in a re-adherent cell.

The number of discontinuous Golgi objects in a 3D de-convoluted image was determined by using the advanced object analysis plugin of the Huygens Professional software. The garbage volume was set as calculated earlier, and a 15% threshold used to determine the number of discontinuous Golgi objects present in a cell. This was done for all cells in each treatment and the average number of Golgi objects determined, which were then compared between treatments to comment on the extent of the Golgi disorganization. Total Golgi volume was measured by addition of the Golgi volume of each object in a cell at a given time or treatment.

### Colocalization analysis

Colocalization analysis of the cis Golgi (GM130), cis-medial (ManII), trans-Golgi (GalTase) and ER markers (KDEL-RFP) was done by using the Colocalization Analyzer plug-in in the SVI Huygens Professional software (version 16.10). De-convoluted cross-section images of the cell expressing both markers were opened with this plug-in and Pearson coefficients were calculated for each cell. Values thus obtained across cells were compared between suspended and re-adherent cells and plotted in a graph. A line plot of the intensities for each marker was made using the Huygens twin slicer plug-in. Fluorescence intensities thus obtained for both markers were plotted using GraphPad Prism and the overlap in their intensities compared using the plot.

### Determining the Golgi distribution profile in a cell population

Cells where the Golgi was labeled for a cis, cis-medial and trans-Golgi were imaged using a confocal microscope; the structure of the Golgi was then observed and classified as organized or disorganized. Representative cross-sectional confocal images of the selected organized and disorganized Golgi phenotypes were acquired, de-convoluted and surface rendered. A minimum of 50 (for bead experiments) and maximum of 200 randomly selected cells were observed in each phenotype population, and their Golgi structure was classified as organized or disorganized. The number of cells in each group was then used to calculate the distribution of organized versus disorganized Golgi (in per cent) in each population for a given time point or treatment. Data from multiple experiments using these percentage values were plotted accordingly.

### Endocytosis of ganglioside GM1 in cells treated with Nocodazole and latrunculin A

To test the role of the cytoskeleton regarding the endocytosis of ganglioside-GM1, serum-deprived adherent cells were pre-treated or not with Nocodazole (10 µM) or latrunculin A (0.5 µM) for 1 h, detached with trypsin and incubated with CTxB conjugated to Alexa Fluor 488 (dilution 1:10,000) for 15 min on ice in low-serum DMEM. These cell surface-labeled cells were washed with low-serum DMEM at RT and fixed with 3.5% PFA in order to obtain 5′ SUSP cells. The remaining GM1-CTxB-labeled cells were held in suspension with 1% methylcellulose (as described above) for 120 min with or without the drug. Post-incubation cells were collected at the required time, carefully washed twice with low-serum DMEM and spun at 367 ***g*** for 5 min at 4°C. Cells were fixed with 3.5% PFA to obtain 120′ SUSP cells.

### Measuring cell volume of suspended and re-adherent cells by surface GM1 labeling

Cells labeled as above and collected when detached (5′ SUSP) or after 120 min in suspension (120′ SUSP) were washed and re-plated on FN-coated (2 µg/ml) coverslips for 5 min and fixed with 3.5% PFA at RT for 15 min. *Z*-stacks of the cells with comparable labeling were collected (0.2 µm cross sections). Images were de-convoluted and surface rendered using the Huygens Professional image analysis software with a 2% threshold setting (see above ‘De-convolution of *z*-stacks and object analysis’). This allowed for the entire volume of the cell to be filled. The volume of each cell was, thus, calculated and compared between suspended and re-adherent cells.

### Binding of FN-beads or PLL-beads to cells

8×10^8^ divinyl polystyrene beads were resuspended in 500 µl PBS and sonicated for 30 s. Beads were washed thrice with cold 1× PBS pH 7.4, centrifuged at 3824 ***g*** for 5 min at 4°C and incubated with 500 µl PBS containing 10 µg/ml FN or 10 µg/ml poly-L-lysine (PLL) at 4°C overnight on a rotary shaker. Coated beads were centrifuged, washed with cold 1× PBS pH 7.4 and blocked with 50 mg/ml BSA at 4°C for 3 h on a rotary shaker. Beads were then washed, resuspended in 100 µl of PBS pH 7.4 and stored at 4°C. Beads were washed with warm PBS and resuspended in 100 μl PBS. Serum-deprived cells (2×10^5^) that had been transfected with GalTase-RFP transfected were detached, held in suspension in 2 ml low-serum DMEM supplemented with 1% methylcellulose for 30 min. FN- or PLL-coated beads in suspension (12.5 µl, 2×10^6^ beads) were added to the cells (with a cell:bead ratio of 1:10) mixed gently and incubated for 15 min at 37°C. Cells were washed gently and fixed with 3.5% PFA for 15 min at RT. Cells were then mounted using Fluoromount-G, allowed to dry for 24 h and imaged. Cells with bound beads were identified and used for comparison.

### Spatial Golgi localization relative to a cell-bound FN-coated bead

Cross-sectional images of cells with cis-Golgi (GM130) or trans-Golgi (GalTase) and a single attached FN bead were selected. The labeled Golgi area was then mapped using a freehand tool in Microsoft PowerPoint, the cell perimeter was marked by a blue circle and the location of the bead with the red circle. The line drawings for cell outline plus bead outline plus mapped Golgi area for each cell were grouped together for each cell. The resulting groupings from multiple cells were adjusted, such that the bead position for all cells overlapped. The relative position of the Golgi for each cell continued to be maintained relative to the bead and was then comparable across cells. This allowed us to generate a combined image of all cells, with their cell perimeter and bead position identical and the Golgi mapped in purple. Golgi outlines are made transparent allowing us to view their overlapping localization.

### Treatment of non-adherent cells with RGD or RGE peptide

WT-MEFs (4×10^5^) transiently expressing GalTase-RFP for 36 h were serum deprived for 12 h, detached using Accutase at 37°C for 1 min, washed and held in suspension for 120 min as described earlier. Cells were then spun down, washed and reconstituted in 200 µl of low-serum DMEM and mock-treated with Milli-Q water (CNT) or incubated with 40 µg/ml RGD peptide or 40 µg/ml RGE peptide for 15 min at 37°C. Cells were fixed with 3.5% PFA for 15 min at RT, mounted using Fluoromount-G, allowed to dry for 24 h and then imaged.

### β1-Integrin blocking antibody (4B4)-mediated inhibition of re-adhesion

Human fibroblast (BJ) cells (4×10^5^) transiently expressing GalTase-RFP for 36 h were serum deprived for 12 h, detached using Accutase and held in suspension for 120 min at 37°C. Cells were then washed and reconstituted in 400 μl of low-serum DMEM and mock treated (CNT) or incubated with 10 µg/ml IgG-mouse antibody or 10 µg/ml β1-integrin function blocking antibody (4B4 clone) for 15 min at 37°C. Cells were then re-plated on coverslips coated with 2 µg/ml FN (with or without the antibody) for 5–10 min and fixed with 3.5% PFA at RT for 15 min. Cells were mounted using Fluoromount-G, allowed to dry for 24 h and then imaged by using a confocal microscope.

### Immunofluorescence staining of cells for γ-tubulin, β-tubulin and actin

#### γ-Tubulin and actin

Serum-deprived cells (4×10^5^) held in suspension or re-adherent on FN for the required time were fixed with 3.5% PFA at RT, permeabilized with PBS containing 5% BSA and 0.05% Triton X-100 for 10 min at RT. Cells were then blocked with 5% BSA in PBS for 30 min at RT and incubated with anti-γ-tubulin antibody diluted 1:100 in PBS with 5% BSA or with phalloidin conjugated to Alexa Fluor 568 diluted 1:400 in 5% BSA for 1 h at RT. Cells were washed with PBS and incubated with anti-mouse Alexa Fluor 488 (diluted 1:1000 with 5% BSA) at RT for 1 h.

#### β-Tubulin

Serum-deprived cells (4×10^5^) held in suspension or re-adherent on FN were fixed by treating cells for 1 min with 100% methanol and kept at −20°C for 1 min. Cells were permeabilized in PBS containing 5% BSA and 0.05% Triton X-100 for 10 min at RT, blocked with PBS containing 5% BSA for 30 min at RT and incubated with anti-β-tubulin antibody (1:1000) in PBS with 5% BSA overnight at +4°C. Cells were washed with PBS and incubated with diluted anti-mouse Alexa Fluor 488 antibody (1:1000) at RT for 1 h.

Suspended cells were similarly labeled in Eppendorf tubes where the reagent volume was always 50 µl (blocking, permeabilization, primary antibody and secondary antibody) and mixed regularly by tapping to prevent cells from settling. Cells were washed with 1× PBS and eventually reconstituted in 10 µl PBS, mounted using Fluoromount-G, allowed to dry for 24 h and imaged by using a confocal microscope.

### Inhibitor treatment in suspended and re-adherent cells

For all experiments using inhibitor, 4×10^5^ cells transiently expressing GalTase-RFP and ManII-GFP were serum deprived for 12 h in low-serum DMEM, detached and held in suspension for 60 min in 5 ml of 1% methylcellulose-containing DMEM at 37°C. Cells were then treated with Nocodazole (10 µM in DMSO), latrunculin A (0.5 µM in DMSO), BFA (10 µg/ml in methanol), Golgicide A (10 µM in DMSO) or Ciliobrevin D (20 µM in DMSO) and incubated for an additional 30 min at 37°C. Control cells were treated with an equivalent volume of solvent (DMSO supplemented with methanol). Cells were processed as described in Materials and Methods under ‘Suspension and re-adhesion of cells’ and samples collected at required times. When needed, cells were re-plated on FN-coated coverslips (processed as described earlier) with or without inhibitor for 5 min, fixed, mounted using Fluoromount-G, allowed to dry for 24 h and then imaged.

### Inhibitor studies – Nocodazole washout assay

Serum-deprived 4×10^5^ WT-MEFs were detached and held in suspension for 60 min and then incubated with Nocodazole (10 µM in DMSO) for 60 min at 37°C. Suspended cells were washed twice (6–8 min) with PBS to remove methylcellulose and Nocodazole from the washed out cells. Nocodazole (10 µM) was added again. Cells with or without Nocodazole were re-plated on FN-coated coverslips for 8 min; one set was fixed with 3.5% PFA and a second set with 100% methanol (at −20°C for 1 min). PFA fixed cells were used to stain for GM130 and methanol-fixed cells stained for β-tubulin as described above. Cells were then mounted using Fluoromount-G, allowed to dry for 24 h and imaged by using a confocal microscope.

### Arf1 activity assay

WT-MEFs (10×10^5^) were serum deprived in low-serum DMEM for 12 h, detached using trypsin-EDTA (5′ SUSP), kept in suspension with 1% methylcellulose for 120 min (120′ SUSP), re-plated on FN (10 µg/ml) for 5 min (5′ FN) or for 4 h to become stable adherent. At the end of incubation times, cells were frozen, lysed in activity assay buffer and activated Arf1 was pulled down using the glutathione S-transferase (GST)-tagged Golgi-localized γ-ear containing Arf-binding protein 3 (GGA3) fusion protein (GST-GGA3) as described earlier (Pawar et al., 2016). The GST-Sec5 Ral-binding domain (Sec5-RBD) (Pawar et al., 2016) was used as negative control for these pulldowns. For pulldown assay, 400 µl of cell lysate was used, which was itself eluted in 20 µl of Laemmli buffer. 22.5 µl (of 400 μl) of whole-cell lysate (2.8% of total) and all of the GGA3 pulldown sample (100% of total) were resolved by 12.5% SDS-PAGE and transferred to PVDF membrane (Millipore). Blots were blocked with Tris-buffered saline containing 5% milk, 0.1% Tween-20 (TBST) for 1 h at RT and incubated at 4°C overnight with the anti-Arf1 antibody (Clone 1D9, Abcam) diluted 1:500 in 2.5% milk in TBST. Blots were washed and incubated with anti-mouse HRP diluted 1:10,000 in 2.5% milk in TBST at RT for 1 h and developed using the PICO chemiluminescence detection system (Thermo Scientific). ImageQuant LAS 4000 (Fujifilm-GE) was used to image the blots; densitometric band analysis was done using ImageJ software (NIH).

#### Arf1 activity assay for inhibitor studies

WT-MEFs (10×10^5^) were serum deprived for 12 h in low-serum DMEM, detached and held in suspension for 60 min in 5 ml of 1% methylcellulose containing low-serum DMEM at 37°C. Cells were then treated with BFA (10 µg/ml in methanol) or Ciliobrevin D (20 µM in DMSO) and incubated for an additional 30 min at 37°C. Control cells were treated with an equivalent volume of solvent (DMSO/methanol). Cells were then washed and lysed, and activated Arf1 was pulled down with GST-GGA3 and detected as described in Materials and Methods under ‘Arf1 activity assay’.

#### Arf1 activity assay for RGD versus RGE peptide treatment studies

10×10^5^ WT-MEFs were serum deprived for 12 h, detached using Accutase at 37°C for 1 min, washed and held in suspension for 120 min as described in the suspension assay protocol. Cells were then centrifuged, washed and reconstituted in 500 µl of low-serum DMEM and mock treated (CNT) or incubated with 40 µg/ml RGD peptide or 40 µg/ml RGE peptide for 15 min at 37°C. Cells were then washed and lysed in Arf1 activity assay buffer, and activated Arf1 was pulled down with GST-GGA3 and detected as described above.

### Detection of dynein associated with activated Arf1 in GGA3 pulldown

WT-MEFs (10×10^5^) were serum deprived in low-serum DMEM for 12 h, detached using trypsin-EDTA (5′ SUSP), kept in suspension with 1% methylcellulose for 120 min (120′ SUSP) and re-plated on FN (10 µg/ml) for 5 min (5′ FN) or 15 min (15′ FN) and for 4 h to be stable adherent). Activated Arf1 was pulled down using the GST-GGA3 as described above for the activity assay. Pulldown and whole-cell lysate samples were probed for mouse anti-Arf1 antibody (clone 1D9, Abcam, cat. no. ab2806) at a dilution of 1:500 and mouse anti-dynein antibody (clone 74.1, Millipore, cat. no. MAB1618) at a dilution of 1:2000. Arf1 and dynein levels in the pulldown were normalized to their respective levels in the whole-cell lysate, values thus obtained were normalized to stable adherent cells and compared suspended and re-adherent cells.

### Cell surface lectin binding and quantitation by flow cytometry

For experiments looking at cell membrane lectin binding, WT-MEFs that had been serum deprived for 12 h were detached (5′ SUSP) using Accutase, washed and held in suspension for 120 min (120′ SUSP). 10×10^5^ live cells following detachment (5′ SUSP) and after 120 min in suspension (120′ SUSP), were incubated with ConA-Alexa 488 (0.025 µg/µl), PNA (0.025 µg/µL), WGA (0.0005 µg/µl) and FITC-UEA (0.1 µg/µl) for 15 min on ice in the dark in 200 µl PBS. Cells were gently mixed during incubation to avoid clumping. They were eventually washed with cold PBS, fixed with 3.5% PFA for 15 min at RT and resuspended in 200 µl PBS. Cells were analyzed using the BD LSRFortessa SORP cell analyzer (BD Bioscience). Unlabeled detached 5′ SUSP and 120′ SUSP cells were analyzed to set forward and side-scatter profiles and this was then used to set a gate to eliminate any autofluorescence from unlabeled cells. Bound lectin fluorescence was then measured as 10,000 events were recorded for each treatment or time point of the experiment. This data was analyzed using the Flowing software 2.5.1 and median fluorescence intensity was calculated for each cell population. Median fluorescence intensities when required were normalized to respective 5′ SUSP time points and compared.

### Kinetics of cell-surface lectin binding

For time kinetics studies, WT-MEFs serum deprived for 12 h were detached using Accutase (Sigma), and 10×10^5^ cells were held in suspension for 5, 10, 20, 30, 60 and 120 min. At the end of each time point, cells were washed and labeled with ConA-Alexa 488 (0.025 µg/µl) for 15 min on ice in the dark in 200 µl PBS. Cells were further processed and fixed as above and analyzed using the BD LSRFortessa SORP cell analyzer as described above. This data was analyzed using the Flowing software 2.5.1 and median fluorescence intensity was calculated for each cell population.

### Effect of CHX treatment on cell surface lectin binding

WT-MEFs serum deprived for 8 h were either mock treated or treated with 20 µg/ml CHX for 4 h under low-serum conditions. Cells were detached with Accutase (5′ SUSP) and held in suspension for 120 min in low-serum DMEM with 1% methylcellulose. Cells were washed after each time point and labeled with ConA-Alexa 488 (0.025 µg/µl) for 15 min on ice in the dark in 200 µl PBS. Cells were fixed and processed using the BD LSRFortessa SORP cell analyzer as described above. 10,000 events were recorded for each time point and data was analyzed using the Flowing software 2.5.1. Median fluorescence intensity calculated for the 120′ SUSP cell population was normalized to the 5′ SUSP cell population in control and CHX treated cells, respectively.

### Cell surface ConA binding in Arf1 mutant-expressing cells

WT-MEFs transfected with empty mCherryN1 (CNT), WT-Arf1-mCherryN1 (WT-Arf1) and Q71L-Arf1-mCherryN1 (Q71L Arf1) using LTX-PLUS transfection reagent (Invitrogen) were serum deprived for 12 h, detached (5′ SUSP) using Accutase and held in suspension for 120 min (120′ SUSP). 10×10^5^ live cells (for each construct) were collected at both time points. Cells were surface labeled with ConA-Alexa 488 (0.025 µg/µl) as described above. 7000 cells in each population gated for mCherry fluorescence (and hence Arf expression) were analyzed for their lectin binding (ConA-Alexa 488). Flowing software 2.5.1 was used to determine the median fluorescence intensity for bound ConA in cells suspended for 120 min (CNT, WT Arf1, Q71L Arf1) and normalized to the median fluorescence intensity of their respective 5′ SUSP samples (equated to 100). Surface-bound ConA levels (5′ SUSP) were also compared across cells expressing Arf1 mCherry constructs, as were the levels of mCherry fluorescence levels for WT-Arf1-mCherry and Q71L-Arf1-mCherry constructs.

### Statistical analysis

All the analysis was done using Prism Graphpad analysis software. Statistical analysis of the number of Golgi objects, Arf1 activation, Pearson coefficient and levels of bound lectin were all done using the Mann–Whitney U test. When data were normalized to a control and compared, one-sample *t*-test was used. Statistical analysis for changes in distribution profile of Golgi phenotype was done using the χ^2^ *t*-test.

## Supplementary Material

Supplementary information
