# Supplementary material for: Cell-matrix adhesion controls Golgi organization and function through Arf1 activation in anchorage-dependent cells
Source: J Cell Sci. 2018 Aug 17;131(16):jcs215855. doi: 10.1242/jcs.215855 (PMC6127727; doi:10.1242/jcs.215855)
Supplement: Supplementary information [file joces-131-215855-s1.pdf]

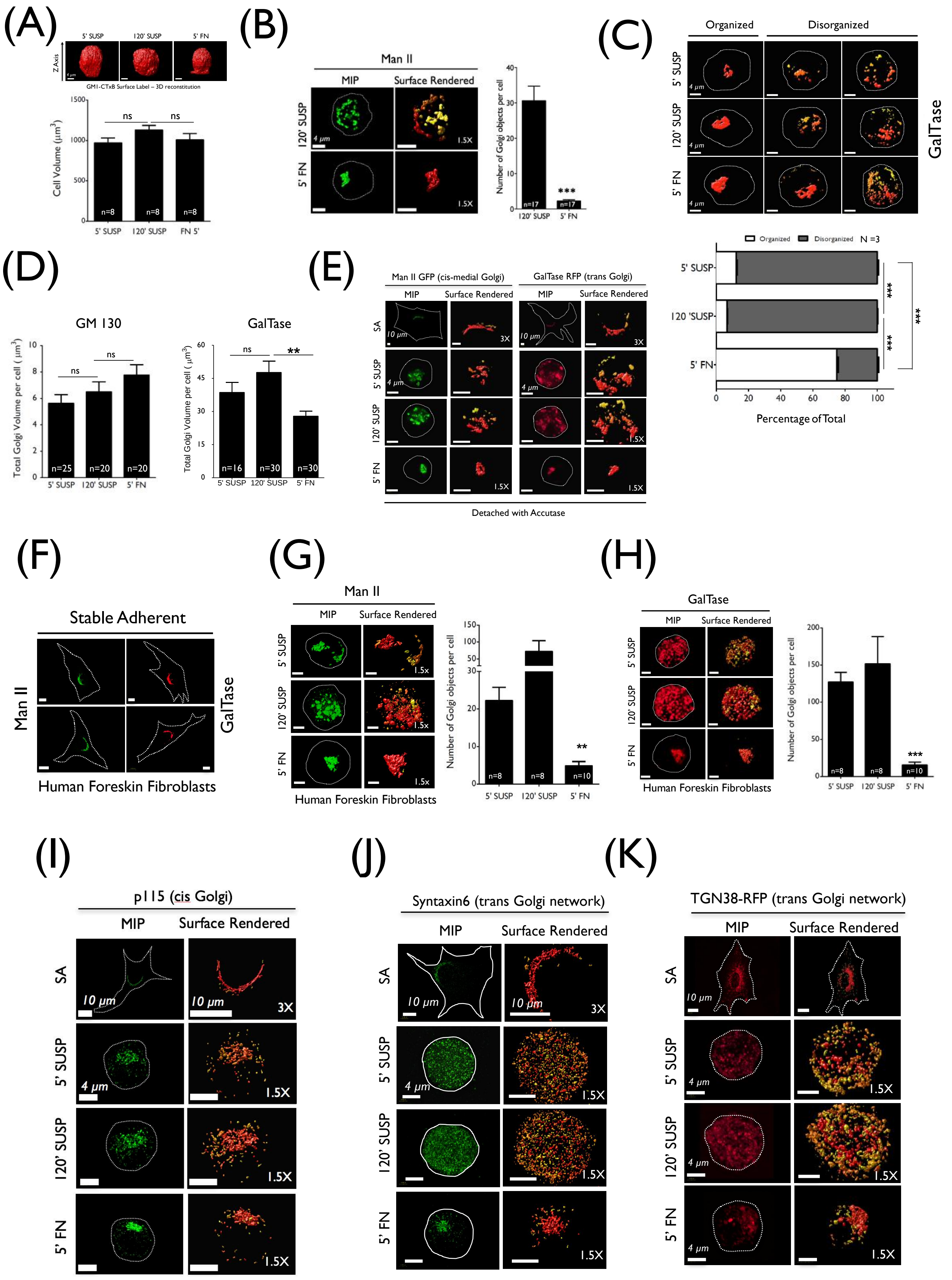
(A)
5' SUSP
120' SUSP
5' FN
Z Axis
GM1-CTxB Surface Label – 3D reconstitution
Cell Volume (μm³)
ns
ns
n=8
n=8
n=8
5' SUSP
120' SUSP
FN 5'
(B)
Man II
MIP
Surface Rendered
120' SUSP
5' FN
4 μm
1.5X
Number of Golgi objects per cell
***
n=17
n=17
120' SUSP
5' FN
(C)
Organized
Disorganized
5' SUSP
120' SUSP
5' FN
GalTase
Organized
Disorganized
N =3
5' SUSP
120 'SUSP
5' FN
***
Percentage of Total
(D)
GM 130
Total Golgi Volume per cell (μm³)
ns
ns
n=25
n=20
n=20
5' SUSP
120' SUSP
5' FN
GalTase
ns
**
n=16
n=30
n=30
(E)
Man II GFP (cis-medial Golgi)
GalTase RFP (trans Golgi)
MIP
Surface Rendered
SA
10 μm
3X
Detached with Accutase
(F)
Stable Adherent
Man II
GalTase
Human Foreskin Fibroblasts
(G)
Man II
n=8
n=8
n=10
(H)
GalTase
(I)
p115 (cis Golgi)
(J)
Syntaxin6 (trans Golgi network)
(K)
TGN38-RFP (trans Golgi network)

Fig. S1. Adhesion dependent Golgi organization detected using Golgi markers. (A) WT-MEFs pre-labeled with GM1-CTxB on ice for 15' were detached (5' SUSP), suspended for 120min (120' SUSP), and re-adherent on fibronectin (5' FN). Confocal Z stacks were deconvoluted, 3D reconstituted and observed along their Z-Axis. Graph represents volume of cells (mean ± SE) of 8 such cells from 2 independent experiments. **(B)** WT-MEFs transfected with ManII were detached and suspended for 120min (120' SUSP) and re-adhered on fibronectin for 5min (5'FN). Representative MIP (left panel) and deconvoluted surface rendered images (right panel) (zoomed 1.5X) are shown. Graph represents discontinuous Golgi objects per cell as mean ± SE from 17 cells from 3 independent experiments. **(C)** Percentage distribution of WT-MEFs with organized and disorganized Golgi phenotypes in cell populations from detached (5' SUSP), suspended (120' SUSP) and re-adherent cells (5' FN) were determined and representative surface rendered cross section images shown. The graph represents mean ± SE from 3 independent experiments. **(D)** The total cis-Golgi (GM130) and trans-Golgi **(**GalTase) volume measured in deconvoluted Golgi Z stack images is represented in the graph (mean ± SE) from 16-30 cells (as indicated in each bar) from 3 independent experiments. **(E)** WT-MEFs expressing ManII (left panel) and GalTase (right panel) were detached using Accutase and stable adherent (SA), detached (5' SUSP), suspended (120' SUSP) and readherent (5' FN) cells were imaged. Representative cross sectional MIP image and 3X (for SA) 1.5 X (for other time points) zoomed surface rendered images are shown from 3 independent experiments. Golgi labelled with **(F)** ManII (left panel) or GalTase (right panel) in stable adherent human foreskin fibroblasts transfected, **(G, H)** detached (5' SUSP), suspended (120' SUSP), and re-adherent (5' FN) were imaged. MIP and surface rendered (1.5X zoomed) de-convoluted confocal z-stack of representative cells with **(G)** Man II and **(H)** GalTase are shown. Graph represents discontinuous Golgi objects per cell as mean ± SE from 8-10 cells from 3 independent experiments. Serum-deprived WT MEFs immunostained for **(I)** cis-Golgi marker (p115), **(J)** trans-Golgi marker (Syntaxin6) and **(K)** transfected with trans-Golgi network (TGN38-RFP) were imaged when stable adherent (SA), detached (5'SUSP), suspended (120'SUSP) and re-adherent (5'FN) time points. Representative MIP (left panel) and deconvoluted surface rendered images (right panel) (zoomed 3X for SA and 1.5x for others) are shown from 3 independent experiments. Scale bars in images are set at 10 µm (SA) or 4 µm (all others). Statistical analysis was done using Mann Whitney's test in all the graphs except for distribution profile where Chi-Square test was performed (** p-value <0.001, *** p-value <0.0001, ns = not significant).

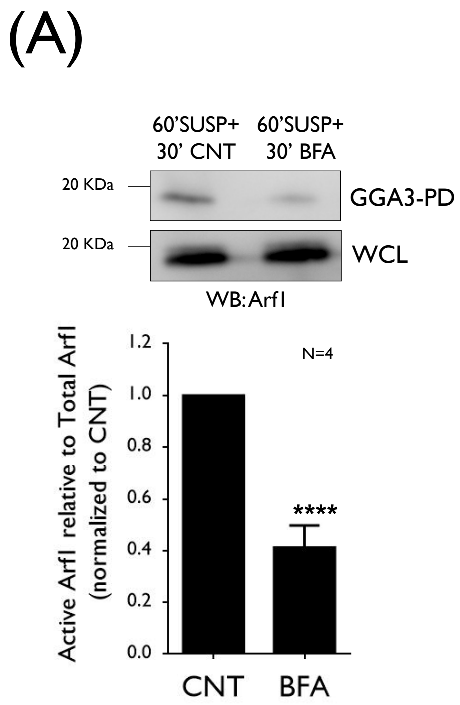

Fig. S2. BFA mediated inhibition of Arf1 activation in non-adherent cells. (A) Western blot detection of active Arf1 (WB: Arf1) pulled down with GST-GGA3 (GGA3 PD) and total Arf1 in whole cell lysate (WCL) from from WT-MEFs suspended for 60min and treated for 30min with methanol (60' SUSP+30' CNT) or 10 µg/ml BFA (60' SUSP + 30' BFA). Ratio of densitometric band intensities of Arf1 in GGA3-PD relative to their levels in the WCL are normalized to control (CNT) represented in the graph as mean ± SE from 3 independent experiments. Statistical analysis was done using the one sample t-test (*** p-value <0.001).

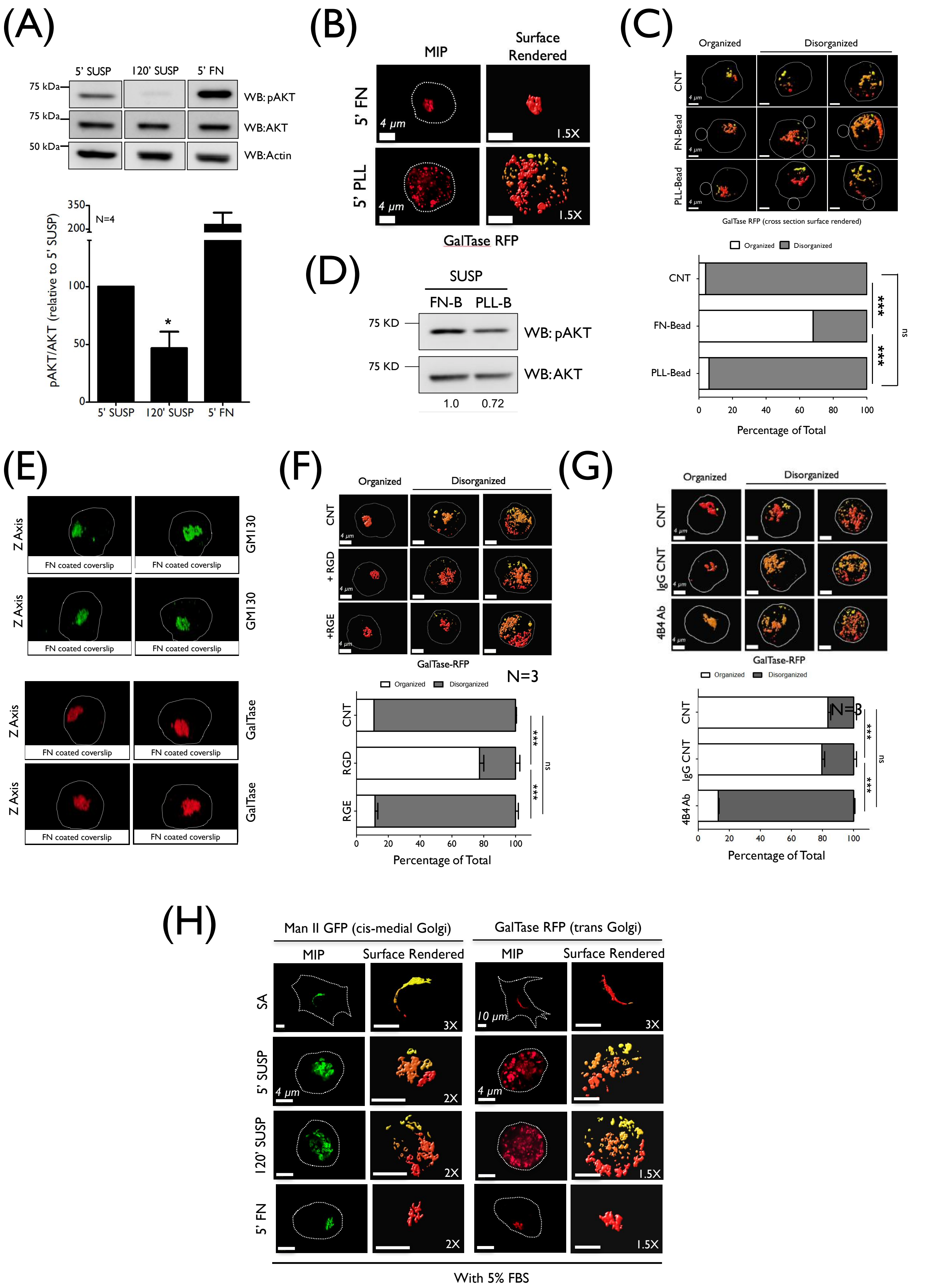

Journal of Cell Science • Supplementary information

Fig. S3. Integrin dependent regulation of Golgi organization. (A) WB detection of AKT phosphorylated at Serine 473 (WB:pAKT) relative to total AKT (WB: AKT) and actin (WB: actin) in lysates from detached (5'SUSP), suspended (120' SUSP) and re-adherent (5' FN) WT-MEFs. Ratio of densitometric band intensities of pAKT relative to AKT in WCL and normalized to ratio in detached cells is represented in the graph as mean ± SE from 4 independent experiments. Statistical analysis was done using the one sample t-test (* p-value <0.01). **(B)** GalTase expressing WT-MEFs suspended for 120 mins and re-plated on fibronectin (5'FN) or poly L-Lysine (5'PLL). Representative MIP and 1.5X zoomed surface rendered images are shown from 3 independent experiments. **(C)** Percentage distribution of WT-MEFs with organized and disorganized Golgi phenotypes in suspended control (CNT), fibronectin coated bead (FN-Bead) bound and poly-l-lysine coated bead (PLL-Bead) bound cell populations were determined and representative surface rendered cross section images shown. The graph represents data from one of two independent experiments with similar outcomes. **(D)** WB detection of AKT phosphorylated at Serine 473 (WB:pAKT) relative to total AKT (WB: AKT) in lysates from suspended FN-Bead (FN-B) or PLL-Bead (PLL-B) bound WT-MEFs. Densitometric band intensities of pAKT relative to AKT in WCL are normalized to ratio in FN-Bead (FN-B) bound cell lysate. Date is representative from 2 independent experiments. **(E)** WT-MEFs suspended for 120' were re-plated on fibronectin for 5 min (5' FN) were immunostained for GM130 (Top panel) or expressing GalTase (Bottom panel). Confocal Z stack images for both were deconvoluted, 3D reconstituted and observed along their Z-Axis relative to the coverslip (FN coated coverslip). Four representative cells for each marker are represented. **(F)** Percentage distribution of cells with organized and disorganized Golgi phenotypes from suspended mock treated (CNT), RGD peptide treated (40 µg/ml) (+RGD) and RGE peptide treated (40 µg/ml) (+RGE) were determined and representative surface rendered cross section images shown. The graph represents mean ± SE from 3 independent experiments. **(G)** Percentage distribution of human fibroblasts (BJ cells) with organized and disorganized Golgi phenotypes in re-adherent mock treated (CNT), mouse IgG treated (10 µg/ml) (IgG CNT) and beta1 integrin blocking 4B4 antibody treated (10 µg/ml) (4B4 Ab) was determined. Representative surface rendered cross section images shown. The graph represents mean ± SE from 3 independent experiments. **(H)** WT-MEFs expressing ManII-GFP and GalTase-RFP were cultured with 5% FBS. Stable adherent (SA) cells were detached (5'SUSP), suspended (120'SUSP) and re-plated on fibronectin (5' FN). Representative MIP and surface rendered (3X for SA and 1.5X for other time points) de-convoluted cross-sectional images as shown from 2 independent experiments. All scale bars in images are 4 µm, except stable adherent cells set at 10 µm. Statistical analysis for distribution profile was done using Chi-Square test was performed (** p-value <0.001, *** p-value <0.0001, ns = not significant).

Journal of Cell Science • Supplementary information

(A)

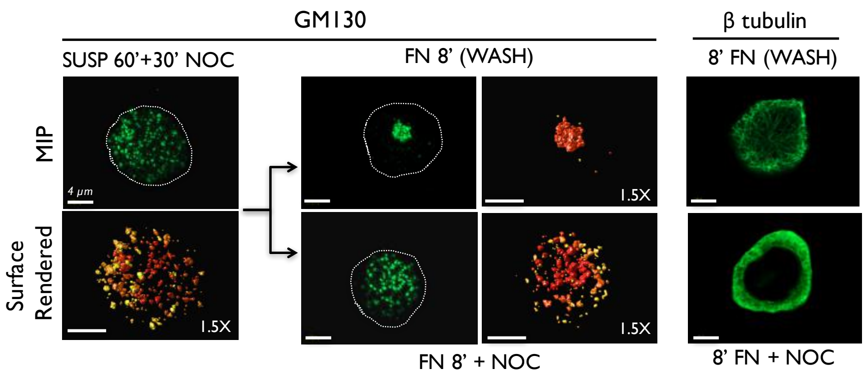

Fig. S4. Nocodazole wash out re-organizes the Golgi. (A) Cells suspended for 60' and then incubated with 10 µM Nocodazole for 30' (SUSP 60' + 30'NOC), were either washed without Nocodazole and re-plated on fibronectin (FN8' - WASH), or re-plated in presence of 10 µM Nocodazole (FN8' + NOC). These cells were immunostained with GM130 and deconvoluted confocal z stacks are represented as MIP and surface rendered images (1.5x zoomed). Beta tubulin stained cells are represented as cross sectional images. Scale bar in all images is set at 4 µm.

Journal of Cell Science • Supplementary information

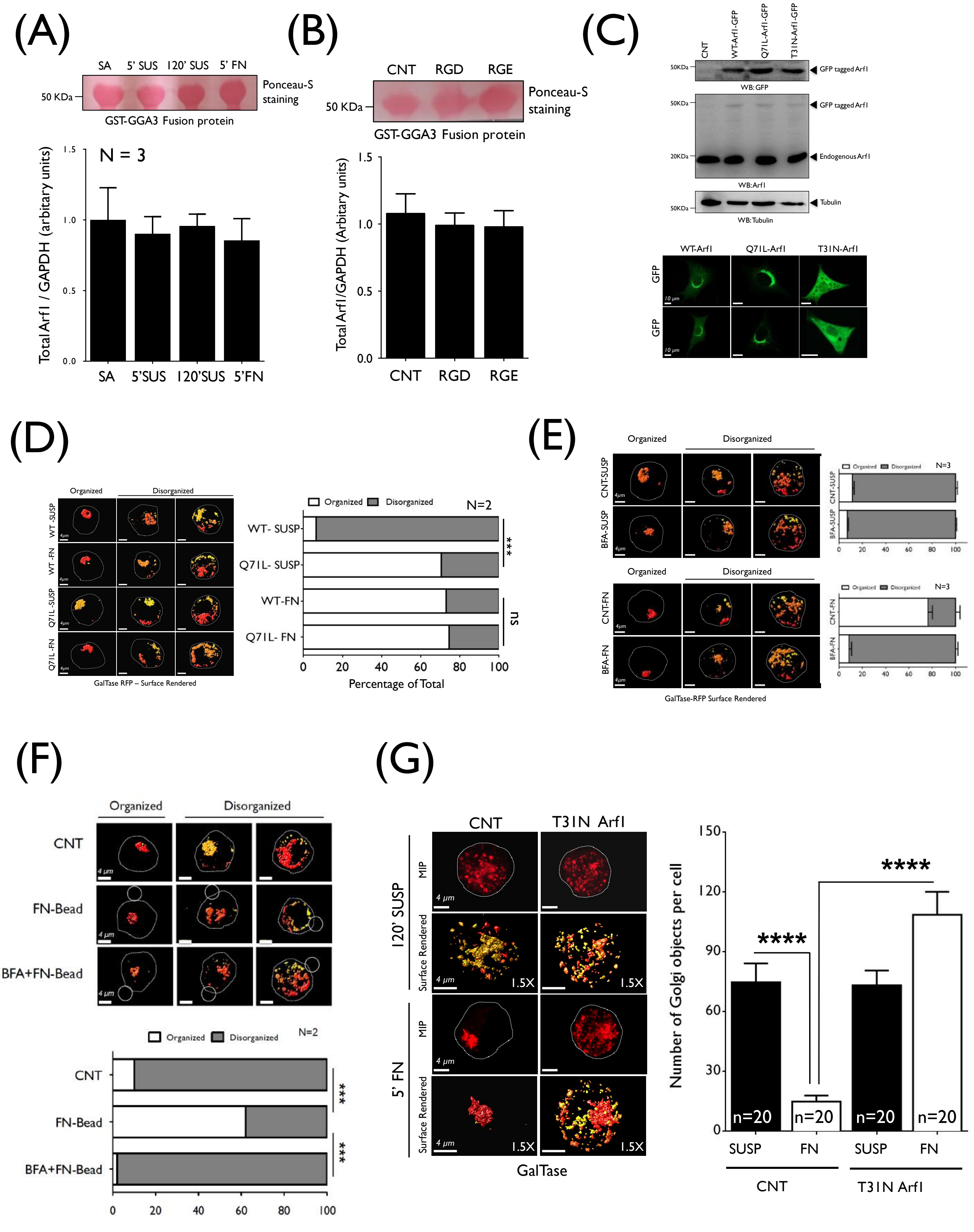

Journal of Cell Science • Supplementary information

Fig. S5. Arf1 activation regulates Golgi organization. (A) Representative Ponceau-S stained blots for GST-GGA3 pulldowns show the GST fusion protein levels being comparable in stable adherent (SA), detached (5' SUSP), suspended (120' SUSP) and re-adherent (5' FN) cell lysate pulldowns. Ratio of densitometric measurement of band intensities of Arf1 and GAPDH detected from western blot of whole cell lysates is represented in the graph as mean ± SE from 3 independent experiments. **(B**) Representative Ponceau-S stained blots for GST-GGA3 pulldowns show the GST fusion protein levels to be comparable in suspended mock treated (CNT), RGD peptide treated (40 μg/ml) (+RGD) and RGE peptide treated (40 μg/ml) (+RGE). Ratio of densitometric measurement of band intensities of Arf1 and GAPDH in WCL detected from western blot of whole cell lysates is represented in the graph as mean ± SE from 3 independent experiments. **(C)** WTMEFs un-transfected (CNT), transfected with GFP-WT-Arf1, GFP-Q71L-Arf1 and GFP-T31N Arf1 were lysed and WCL probed with anti-GFP (WB: GFP), anti Arf1 antibody (WB: Arf1) to detect the endogenous Arf1 (Endogenous Arf1) and overexpressed Arf1 (GFP tagged Arf1) and compared to tubulin (WB: tubulin). Localization of GFP-WT-Arf1, GFP-Q71L-Arf1 and GFP-T31N Arf1 in two representative stable adherent cells are shown (bottom panel). **(D)** Percentage distribution of cells with organized and disorganized Golgi phenotypes from suspended WT Arf1 (WT-SUSP), active Arf1 (Q71L-SUSP), re-adherent WT Arf1 (WT-FN) and active Arf1 (Q71L-FN) expressing cells was determined and representative surface rendered cross section images shown. The graph represents one of two independent experiments which gave similar results. **(E)** Percentage distribution of cells with organized and disorganized Golgi phenotypes from cells suspended for 90min and treated for 30min with methanol (CNT-SUSP) or BFA (BFA-SUSP) and re-adherent on fibronectin for 5min (CNT-FN, BFA-FN) were determined and representative surface rendered cross section images shown. The graph represents mean ± SE from 3 independent experiments. **(F)** Percentage distribution of suspended WT-MEFs with organized and disorganized Golgi phenotypes from control (CNT), FN-Bead bead bound and BFA treated WTMEFs bound to FN-Bead (BFA+FN-Bead) was determined and representative surface rendered cross section images shown. The graph represents one of two independent experiments. **(G)** WT-MEFs expressing GalTase RFP alone (CNT) or with GFP-T31N-Arf1 (T31N Arf1) were suspended (120'SUSP) and re-plated on fibronectin (5'FN). Representative MIP and surface rendered de-convoluted confocal z-stack (zoomed 1.5x) are shown. Graph represents discontinuous Golgi objects per cell as mean ± SE from 20 cells from 3 independent experiments. All scale bars in images are 4 μm, except stable adherent cells set at 10 μm. Statistical analysis was done using Mann Whitney's test in all the graphs except for distribution profile where Chi-Square test was performed (** p-value <0.001, *** p-value <0.0001, ns = not significant).

Journal of Cell Science • Supplementary information

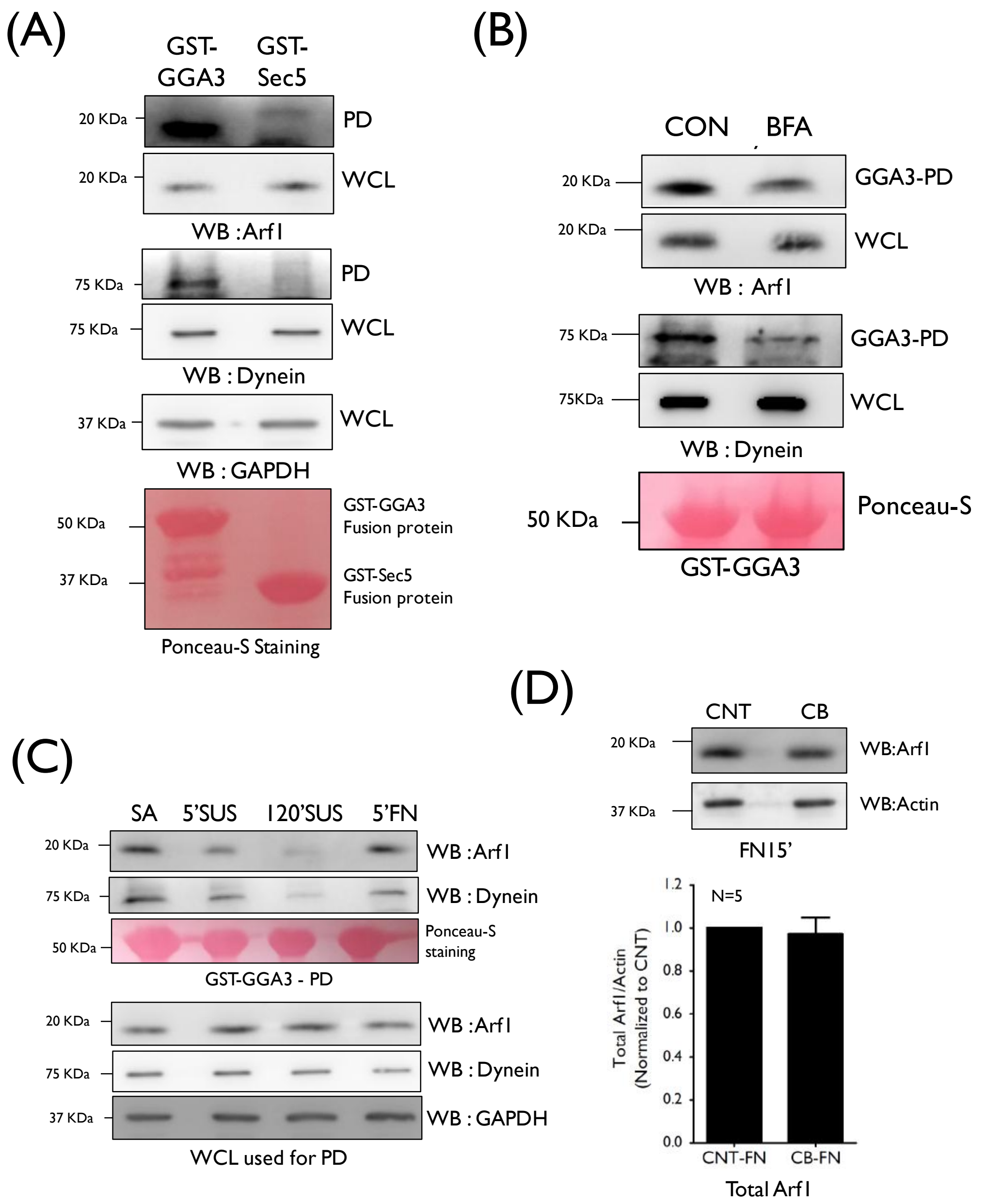

Fig. SF6. Active Arf1 pulldown and its association with dynein. (A) Western blot detection of active Arf1 (WB: Arf1), dynein (WB: Dynein) and GAPDH (WB : GAPDH) in GST-GGA3 and GST-Sec5 pulldowns (PD) and whole cell lysate (WCL) of stable adherent cells . Ponceau-S staining of blots shows GST-GGA3 and GST-Sec5 fusion protein band in the pulldown samples. **(B)** WB detection of active Arf1 (WB : Arf1) and dynein (WB: Dynein) in GGA3 pulldowns (GGA3-PD) from whole cell lysates (WCL) of stable adherent WT-MEFs treated with DMSO (CON) or 10 µg/mL BFA (BFA). Ponceau-S staining of blots shows GST-GGA3 fusion protein band detected in the pulldowns. **(C)** Western blot detection of active Arf1 (WB: Arf1) and dynein (WB:Dynein) in GST-GGA3 pulldowns (GGA3 PD) and total Arf1 & dynein in whole cell lysate (WCL) from stable adherent (SA), detached (5' SUSP), suspended (120' SUSP) and re-adherent (5' FN) WT-MEFs. Ponceau-S staining of pulldown blots detects the GST-GGA3 fusion protein. Blots are representative of four independent experiments. **(D)** WB detection of total Arf1 (WB: Arf1) and Actin (WB: Actin) in WCL of WT-MEFs re-adherent on fibronectin (15' FN) in the absence (CNT) or presence of 20 µM Ciliobrevin (CB). The ratio of densitometric band intensities of total Arf1/Actin is represented in the graph (mean ± SE) from 5 independent experiments.

Journal of Cell Science • Supplementary information

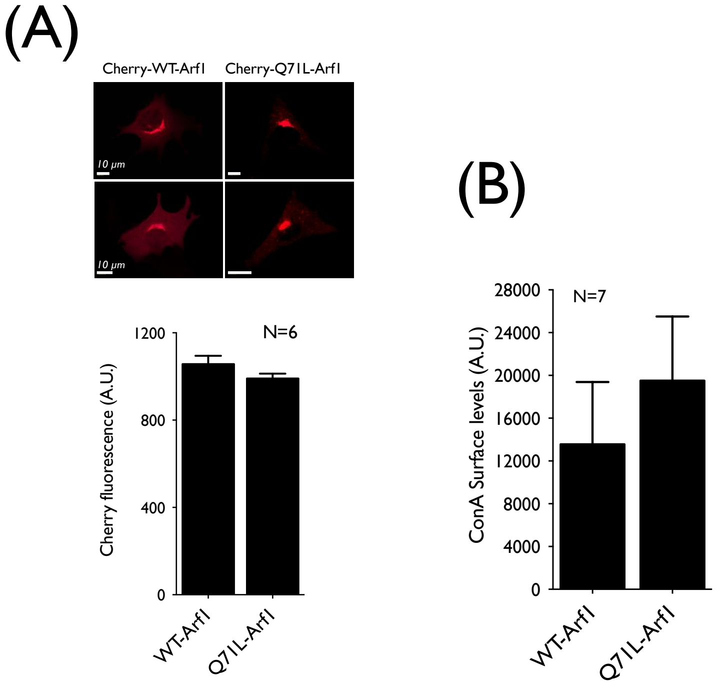

Fig. S7. Expression of Arf1 constructs in WTMEFs. (A) Cherry tagged WT-Arf1 and active Q71L-Arf1 construct expressed in stable adherent (SA) WT-MEFs. The fluorescence intensity of cherry constructs confirming expression of both constructs in cells was analyzed by flow cytometry and median fluorescence intensity represented as in the graph (mean ± SE) from 6 independent experiments. **(B)** Cherry tagged WT-Arf1 or Q71L-Arf1 were detached using Accutase and surface labeled with ConA-Alexa 488. Fluorescence intensity of cell surface bound lectins were measured by flow cytometry in detached (5'SUSP) cell population gated for Cherry tagged Arf1 fluorescence. Average median fluorescence surface ConA fluorescence intensity is represented in the graph (mean ± SE) from 6 independent experiments.

Journal of Cell Science • Supplementary information
